# Mathematical Insights into the Effects of Levodopa

**DOI:** 10.3389/fnint.2012.00021

**Published:** 2012-07-04

**Authors:** Michael C. Reed, H. Frederik Nijhout, Janet A. Best

**Affiliations:** ^1^Department of Mathematics, Duke UniversityDurham, NC, USA; ^2^Department of Biology, Duke UniversityDurham, NC, USA; ^3^Department of Mathematics, Ohio State UniversityColumbus, OH, USA

**Keywords:** Parkinson’s disease, dopamine, serotonin, levodopa, mathematical model

## Abstract

Parkinson’s disease has been traditionally thought of as a dopaminergic disease in which cells of the substantia nigra pars compacta (SNc) die. However, accumulating evidence implies an important role for the serotonergic system in Parkinson’s disease in general and in physiological responses to levodopa therapy, the first line of treatment. We use a mathematical model to investigate the consequences of levodopa therapy on the serotonergic system and on the pulsatile release of dopamine (DA) from dopaminergic and serotonergic terminals in the striatum. Levodopa competes with tyrosine and tryptophan at the blood-brain barrier and is taken up by serotonin neurons in which it competes for aromatic amino acid decarboxylase. The DA produced competes with serotonin (5HT) for packaging into vesicles. We predict the time courses of LD, cytosolic DA, and vesicular DA in 5HT neurons during an LD dose. We predict the time courses of DA and 5HT release from 5HT cell bodies and 5HT terminals as well as the changes in 5HT firing rate due to lower 5HT release. We compute the time course of DA release in the striatum from both 5HT and DA neurons and show how the time course changes as more and more SNc cells die. This enables us to explain the shortening of the therapeutic time window for the efficacy of levodopa as Parkinson’s disease progresses. Finally, we study the effects 5HT1a and 5HT1b autoreceptor agonists and explain why they have a synergistic effect and why they lengthen the therapeutic time window for LD therapy. Our results are consistent with and help explain results in the experimental literature and provide new predictions that can be tested experimentally.

## 1 Introduction

Symptoms of Parkinson’s disease (PD), such as tremor and bradykinesia, arise following degeneration of dopaminergic cells within the substantia nigra pars compacta (SNc), depleting dopamine levels in the basal ganglia. Administration of the dopamine precursor levodopa (LD) has long been the first line of treatment for PD; for many patients, LD therapy successfully relieves symptoms for several years following the initial diagnosis. However, within 5 years of chronic LD treatment, many patients experience a variety of complications (Mouradian et al., 1988). For instance, the length of the therapeutic time window in which a given LD dose relieves PD symptoms gradually shortens and approaches the plasma half-life of LD (wearing-off). Rapid variations in efficacy may occur (on-off fluctuations). Another, particularly troubling, complication of chronic LD therapy is the appearance of involuntary movements (levodopa-induced dyskinesia, LID). These complications increase patients’ disability substantially, posea therapeutic dilemma, and limit the use of LD.

Traditionally, PD has been regarded as primarily a dysfunction of the dopaminergic system in which dopaminergic cells of the SNc die thereby reducing the amount of dopamine (DA) delivered to the striatum. However, recent evidence suggests that the interplay between the serotonergic and dopaminergic systems is critical both for some symptoms of PD and for understanding the side-effects of chronic LD therapy. The raphe nuclei (RN) provide dense serotonergic innervation of the striatum, and the basal ganglia projects back to the RN (Monti, 2010); such reciprocal projections between basal ganglia and raphe nuclei provide a physical substrate for serotonergic involvement in movement. Interestingly Brooks (2007) reports that in order to generate tremors with the characteristic PD frequency of 3–5 Hz in animal models it is necessary to lesion not just nigro-striatal dopaminergic projections but also the midbrain tegmentum, which contains serotonergic cell bodies of the median raphe. He further notes that loss of midbrain serotonin 5HT1a autoreceptors correlates with tremor severity in PD, unlike loss of striatal dopaminergic function. Kish et al. (2008) report that loss of serotonin (5HT) in the striatum is typically both less severe and more variable than dopamine depletion. The variable extent of striatal serotonin loss may reflect competing effects such as raphe cell loss (Jellinger, 1991), molecular regulatory changes (Kish et al., 2008), and compensatory sprouting of 5HT terminals (Maeda et al., 2003).

To understand the interesting and complicated relationship of LD to the serotonergic system, it is useful to briefly sketch the biochemistry. Cells import tyrosine, tryptophan, and other large neutral amino acids by the L-transporter (Kilberg and Haussinger, 1992), which also imports LD when it is in the extracellular space. DA neurons express tyrosine hydroxylase (TH) that converts tyrosine to LD, which is decarboxylated by amino acid decarboxylase (AADC) and converted to DA. Most DA does not remain in the cytosol but is packaged into vesicles by the vesicular monoamine transporter (VMAT). Since DA does not cross the blood-brain barrier (BBB), its precursor, LD, is given instead, with the intent that it will be taken up by DA neurons, converted to DA, and increase the release of DA by the remaining SNc neurons that project to the striatum. In contrast, 5HT neurons express tryptophan hydroxylase (TPH) that converts tryptophan to 5-hydroxytryptophan (5HTP) that is decarboxylated by AADC to form 5HT, which is then packaged into vesicles by VMAT. Thus, it is the differential expression of TH and TPH that makes neurons into DA neurons or 5HT neurons, respectively (Feldman et al., 1997). LD interferes with this distinction because it is taken up by 5HT neurons. And since the 5HT neurons express AADC and VMAT, the LD in 5HT neurons is converted to DA, packaged into vesicles, and released when actions potentials arrive at 5HT terminals. As we will see below, LD outcompetes 5HTP for AADC and DA outcompetes 5HT for VMAT. The net result is that, during an LD dose, 5HT neurons release a substantial amount of DA.

There is a lot of evidence that supports this scenario. Experiments have verified that serotonergic cells can store and release DA *in vivo* and *in vitro* (Nicholson and Brotchie, 2002). Tanaka et al. (1999) showed that, in levodopa treatment of a hemiparkinsonian rat, striatal extracellular DA decreased substantially when the serotonergic system was lesioned. Glial cells also express AADC and so could contribute to the conversion of LD to DA; however, experiments by Kannari et al. (2000), in which they used reserpine to block vesicular packaging, showed a great reduction of extracellular DA, suggesting that most of the levodopa-derived DA is released by exocytosis of vesicles rather than by glia, at least at physiological levels of levodopa administration. Lindgren et al. (2010) showed that 5HT1a autoreceptor agonists (that decrease RN firing) and 5HT1b autoreceptor agonists (that decrease release at 5HT terminals) both lower extracellular DA in the striatum in a dose-dependent manner after an LD dose.

In addition, there is good evidence that large pulses of extracellular DA are the proximal cause of the levodopa-induced dyskinesias (LID) that are seen after long-term dosing with LD (de la Fuente-Fernandez et al., 2001). Carta et al. (2007) have provided conclusive evidence that these large pulses of DA are caused by DA release from serotonergic cells in LID. They showed that either toxic lesion of the serotonergic system or pharmacological impairment of the system with selective serotonin autoreceptor (5HT1a and 5HT1b) agonists results in a nearly complete elimination of LID.

All of these considerations suggest that understanding the dynamics of LD and DA in 5HT neurons and the interactions between 5HT neurons and DA neurons in the striatum during an LD dose are interesting scientific questions with important medical consequences. We have previously constructed mathematical models of a DA terminal (Best et al., 2009) and a 5HT terminal (Best et al., 2010b) and used the models to explore regulatory mechanisms, to explain data, and to formulate and test hypotheses (Best et al., 2009, 2010a, 2011). We have now combined those models and added a model of the RN cell body. Details of the model can be found in the Appendix. In this paper, we use the new model to explore the dynamics of 5HT and DA in 5HT neurons during an LD dose, and to investigate the dynamics of extracellular DA in the striatum. We will see how the dynamics change as more cells in the SNc die and we will see why the therapeutic time window shortens. Finally, we investigate the effects of 5HT1a and 5HT1b agonists and show that they decrease the height of the DA pulse and that they lengthen the therapeutic time window.

## 2 Materials and Methods

**Figure 1:**
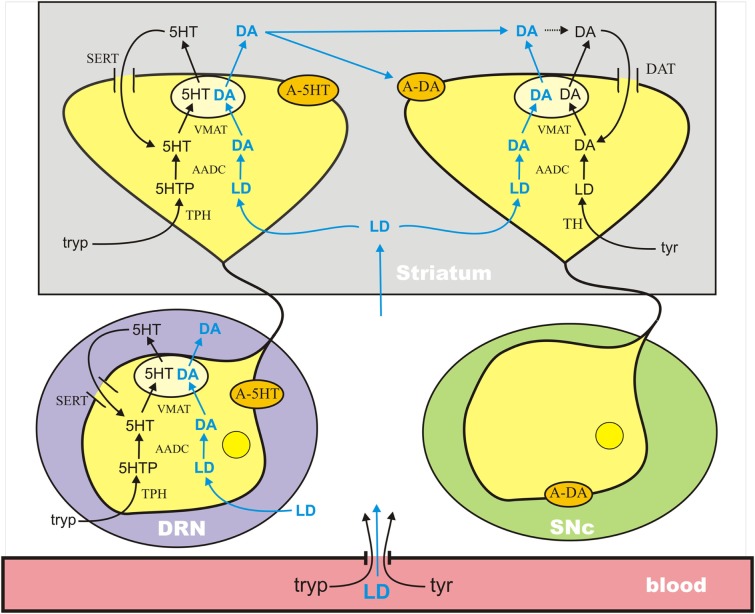
**A schematic diagram of the major components of the mathematical model**. The model combines previously published models of a dopaminergic terminal (Best et al., 2009) and a serotonergic terminal (Best et al., 2010b) in the striatum and adds a model for the serotonergic cell body in the DRN. See the Discussion in the text. A-5HT and A-DA indicate 5HT and DA autoreceptors, respectively. Full details are available in the Appendix.

The mathematical model used in this paper combines previous mathematical models of dopaminergic (Best et al., 2009) and serotonergic (Best et al., 2010b) terminals and adds a new model of the serotonergic cell body in the dorsal raphe nucleus (DRN); see Figure 1. The dopaminergic terminal model includes: transport of tyrosine across the BBB and into the terminal; synthesis of LD by tyrosine hydroxylase (TH), synthesis of cytosolic DA by AADC, packaging of cytosolic DA into vesicles by VMAT, release of vesicular DA into the extracellular space depending on firing rate, reuptake of extracellular DA into the cytosol by the dopamine transporters (DATs), diffusion of extracellular DA out of the system, catabolism of DA in both the extracellular space and the cytosol by monoamine oxidase (MAO), and the effects of extracellular DA on DA synthesis and release via the autoreceptors. The serotonergic model has the same components except that tryptophan and tryptophan hydroxylase (TPH) appear instead of tyrosine and TH, the DATs are replaced by serotonin transporters (SERTs), and many of the detailed rate constants are different.

The mathematical model of the DRN cell body is similar but has important differences from the 5HT terminal model. The biochemistry of synthesis of 5HT in the cell body is the same as in the terminal. However, these 5HT neurons in the DRN have the interesting property that, when they fire, 5HT is released into the extracellular space about the cell body and it is also released by diffusion in the absence of firing (Adell et al., 2002). The extracellular 5HT concentration increases the firing rate when it is low and decreases the firing rate when it is high via the 5HT1a autoreceptors. The firing rate at the cell body affects, of course, the release of 5HT at the terminal. The extracellular concentration of 5HT in the striatum affects synthesis and release at the terminal via the 5HT1b autoreceptors.

Tyrosine, tryptophan, and LD are transported across cell membranes by the L-transporter (Kilberg and Haussinger, 1992), which means that the three substrates compete for transport across the BBB and for entry into cell bodies and terminals. In addition, in 5HT cell bodies and terminals, 5-hydroxytryptamine and LD compete for AADC and 5HT and DA compete for VMAT. Our full model includes the transport of LD across the BBB and the competition for AADC and VMAT in the 5HT neurons. Full details of the mathematical formulas that express these competitions can be found in the Appendix.

## 3 Results

In Section 3.1 we describe the effects of LD on a 5HT neuron. In Section 3.2 we discuss the passive stabilization of extracellular dopamine in the striatum as more and more SNc cells die. In Section 3.3 we investigate how the increase of extracellular DA in the striatum after an LD dose depends on the fraction of SNc cells left alive. In Section 3.4 we discuss the effects of 5HT1a and 5HT1b agonists combined with an LD dose.

### 3.1 The effects of LD on 5HT neurons

#### 3.1.1 Competition at the blood-brain barrier

**Figure 2:**
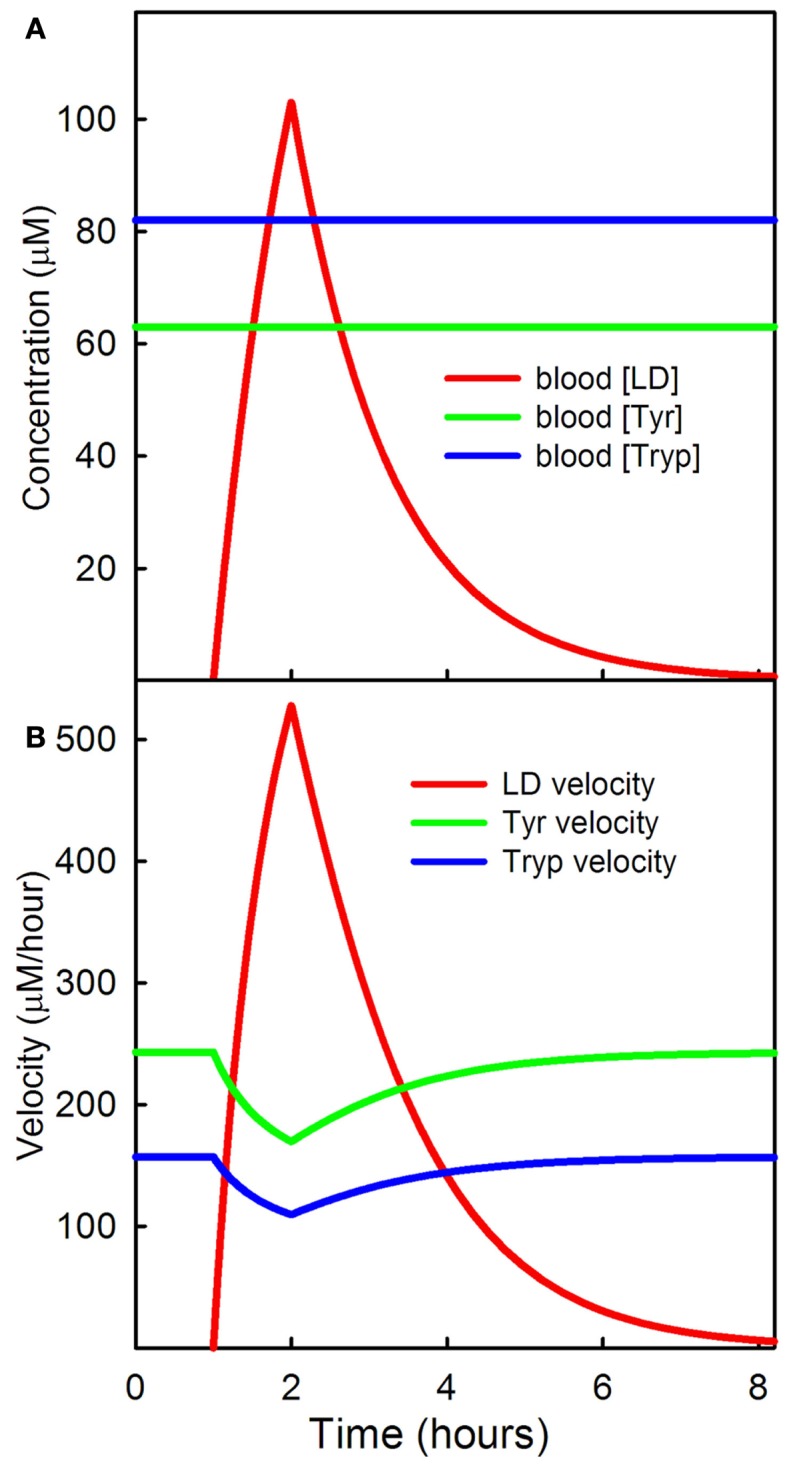
**LD competition with tyrosine and tryptophan at the BBB**. **(A)** Shows the serum levels of a dose of LD; the concentrations of tyrosine and tryptophan are assumed constant. **(B)** Shows the transport velocities of LD, tyrosine, and tryptophan, across the BBB as a function of time.

**Figure 3:**
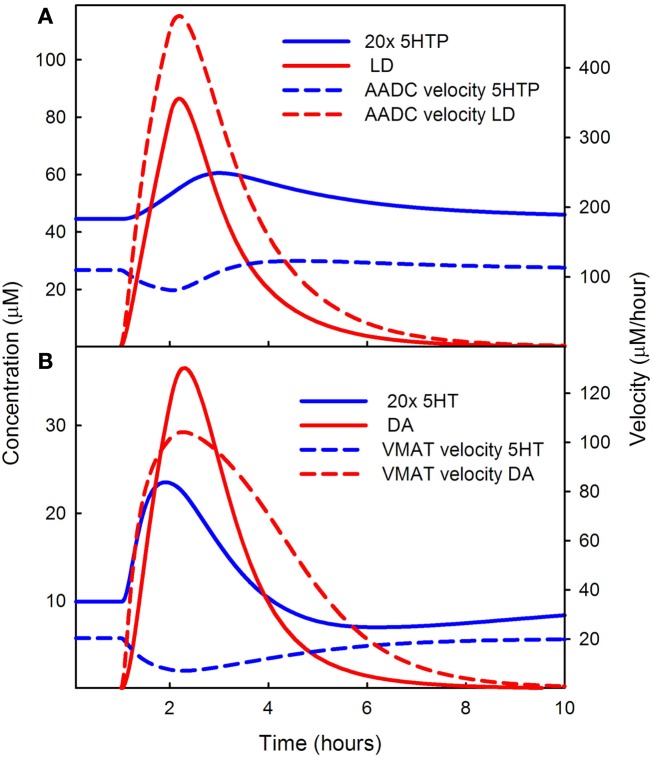
**Competition of LD for AADC and VMAT**. In **(A)** the solid blue curve shows the concentration of 5HTP and the dashed blue curve shows the rate at which 5HT is being produced during the LD dose. The solid red curve shows the concentration of LD in the cytosol and the dashed red curve shows the rate of formation of DA in the 5HT neuron. Note that the solid blue curve blue has been multiplied by an extra factor of 20. LD outcompetes 5HTP for AADC because its concentration in the cytosol is much higher than the concentration of 5HTP. **(B)** Shows 20 times the cytosolic concentration of 5HT (solid blue curve) and the cytosolic concentration of DA (solid red curve). The rate of putting DA into vesicles (dashed red curve) is much higher than the rate of putting 5HT into vesicles. See Discussion in the text.

LD, tyrosine, and tryptophan are transported across the BBB and into the extracellular space and from the extracellular space into neurons and other brain cells by the L-transporter (Kilberg and Haussinger, 1992). The amino acids and LD compete for the transporter and the affinities of the substrates for the transporter are different. The net result is that an increase of one of the substrates in the serum will decrease the transport of the others across the BBB. Figure 2A shows the effect in the model of an LD dose on serum levels of LD where we are assuming that the serum levels of tyrosine and tryptophan are constant in the serum at their normal values. LD has a half-life of about 90 min in the serum and the curve in Figure 2A is similar to the experimental curves in Figures 2 and 3 in the paper of Khor and Hsu (2007), where carbidopa was given with levodopa.

Figure 2B shows the velocities of transport of LD, tyrosine, and tryptophan across the BBB as a function of time during the dose. Because of the competition with LD, the transport of tyrosine and tryptophan decrease during the LD dose. In the rest of this Section we show the downstream results in 5HT neurons of this LD dose.

Conversely, a protein meal simultaneous with or after an LD dose will decrease the import of LD into the brain (simulations not shown). If the transport of LD into the brain varies, one would expect that extracellular DA in the striatum would vary. It was proposed (Nutt, 1987; Pincus and Barry, 1987) as early as 1987 that such variation may be one of the causes of the on-off fluctuations seen clinically in some patients.

#### 3.1.2 Competition for AADC and VMAT

When neurons import LD, the synthesis steps with TH or TPH are bypassed and the enzyme AADC will convert LD into DA in both 5HT and DA neurons (see Figure 1). In 5HT neurons, the cytosolic DA will compete with cytosolic 5HT for VMAT, which packages 5HT and DA into vesicles. For reasons that we will make clear, LD outcompetes 5HTP for AADC and DA outcompetes 5HT for VMAT. The result is that during the LD dose, and for some time thereafter, the vesicles in 5HT neurons contain substantial amounts of DA and when the 5HT neuron fires, DA is released as well as 5HT. In other words, during the LD dose, the 5HT neurons become (partially) DA neurons.

The red solid curve in Figure 3A shows the cytosolic concentration of LD in the model. The concentration of 5HTP (the solid blue curve – note the extra factor of 20) is much lower. The intuitive reason for this is as follows. Normally, the concentrations of 5HTP in 5HT neurons and LD in DA neurons are quite low because they have to be manufactured by THP and TH, respectively, and are quickly decarboxylated. But in supplying LD in the extracellular space at roughly the concentrations of extracellular tyrosine and tryptophan, the synthesis step is omitted. LD is imported by the L-transporter directly and will have quite high intracellular concentrations (as seen in Figure 3A). The *K_m_* values of AADC for 5HTP and LD are roughly comparable 160 and 130 μM, respectively), so the velocities are determined mostly by the large difference in concentration between LD and 5HTP during the dose. Indeed, the velocity at which cda is being made by AADC (dashed red curve) in the 5HT neuron is much greater than the velocity at which 5HT is being made (the dashed blue curve). One can see from Figure 3A that the velocity at which 5HT is being made dips during the LD dose because of the competition with LD.

Because LD outcompetes 5HTP for AADC, during the LD dose, the concentration of cytosolic DA in the 5HT neuron is much greater than the concentration of cytosolic 5HT in the 5HT neuron. This can be seen by comparing the solid red (DA) curve to the solid blue (5HT) curve in Figure 3B. Note that the solid blue curve is 20 times the concentration of cytosolic 5HT. Because of this large difference in concentration, DA outcompetes 5HT for VMAT, the next step in the pathway as shown by the velocities of VMAT for the two substrates in the dashed red and dashed blue curves in Figure 3B. This is true even though the *K_m_* of 5HT for VMAT is quite a bit lower than the *K_m_* of DA for VMAT (0.2 and 3 μM, respectively). As can be seen in Figure 3B, the rate at which 5HT is put into vesicles (the dashed blue curve) dips during the LD dose because of the competition with DA.

#### 3.1.3 Effect on DA and 5HT in vesicles and 5HT in the striatum

**Figure 4:**
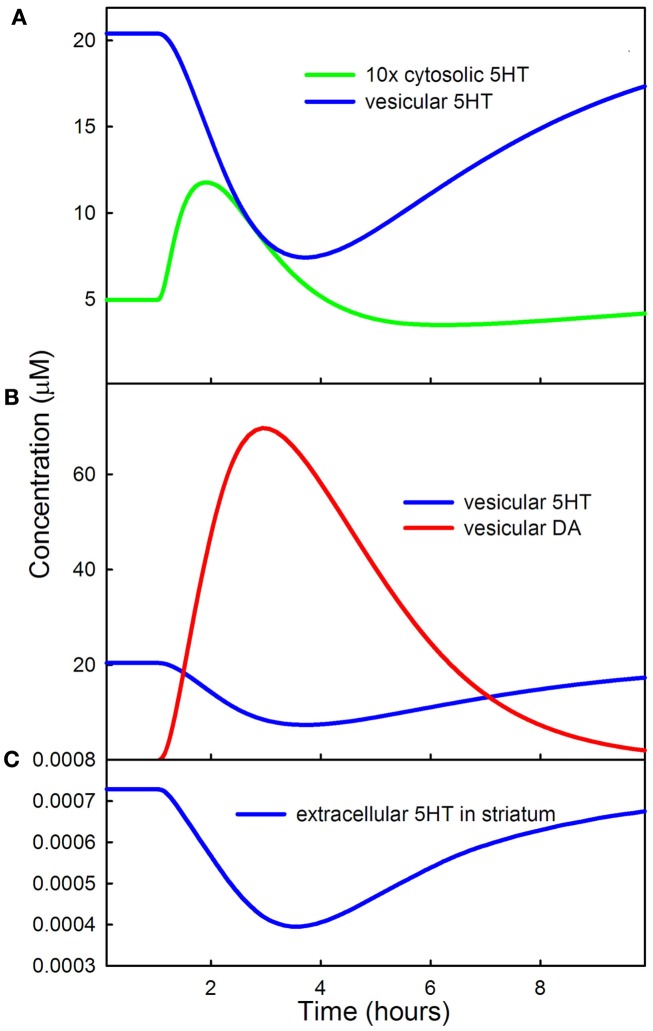
**Effects of LD on cytosolic, vesicular, and extracellular 5HT**. **(A)** Shows that vesicular 5HT declines by more than 50% during the LD dose because of the competition of 5HT and DA for VMAT. The initial rise in cytosolic 5HT is caused by backflow from the vesicles as vesicular input of 5HT declines. **(B)** Shows that DA concentration in the vesicles during the dose is much greater than 5HT concentration. **(C)** Shows the decline of extracellular 5HT in the striatum during the LD dose.

Because the rate at which 5HT is put into vesicles decreases during the LD dose, one would expect that vesicular 5HT would also decrease. This is indeed the case as shown by the blue curve of Figure 4A. Notice that there is a substantial increase in cytosolic 5HT (the green curve) near the beginning of the LD dose. There are two reasons for the increase. First, cytosolic 5HT is being removed and put into vesicles more slowly. Secondly, the rate at which VMAT puts 5HT into vesicles is partly balanced by diffusion of 5HT from the vesicles back into the cytosol. We have included this diffusive backflow in the 5HT neuron because it is known to occur in DA neurons efloor95, wallace07. As the forward rate of VMAT for 5HT drops, backflow from the vesicles contributes to the temporary rise in cytosolic 5HT.

Figure 4B shows the concentrations of DA (red curve) and 5HT (blue curve) in the vesicular compartment of the 5HT terminal during the LD dose. A consequence of LD outcompeting 5HTP for AADC and DA outcompeting 5HT for VMAT, as described above, is that during the LD dose more than 80% of the neurotransmitter in the vesicular compartment of 5HT neurons is DA. Thus, the 5HT neurons are releasing substantially more DA than 5HT.

Figure 4C shows that the concentration of extracellular 5HT in the striatum (or any other projection region) decreases by about 45% in the model during the LD dose. This is consistent with experimental results. Borah and Mohanakumar (2007) measured extracellular 5HTin different brain regions during a dose of LD and found decreases in the range of 50–90% depending on region. Tissue 5HT gives an indirect measure of extracellular 5HT because the amounts released are proportional to storage in vesicles. Carta et al. (2007) found that tissue 5HT decreases 48% in the striatum during an LD dose and Navailles et al. (2011) showed that tissue 5HT decreases 30% in the striatum and 53% in motor cortex during chronic LD dosing and that 5-HIAA (a metabolite of 5HT) decreases by 32.

Decreased serotonergic signaling has been linked to depression (Mann, 1999), so the observation that LD lowers extracellular 5HT concentrations raises the question of whether LD therapy might lead to depression. Indeed, acute tryptophan depletion, known to lower 5HT levels in various brain levels in animals (Moore et al., 2000), results in lowered mood in humans (Young et al., 1985). Given the large decreases in brain 5HT levels described above, one must expect effects on the functioning of the 5HT system; however, many complicating factors make it difficult to draw conclusions about possible connections between LD therapy and depression (Pålhagen et al., 2008; Frisina et al., 2009). Depression is frequently described as the most common psychiatric complication in PD (Lemke, 2008), though reported rates vary widely, from 2.7% to greater than 90% (Reijnders et al., 2008), due to factors including whether both major and minor depression are included and how subjects are selected for inclusion in the study. Some authors have considered the extent to which depression may occur in reaction to the burden of this chronic disease, finding that younger patients, for whom PD may threaten career and life trajectory, are more vulnerable to depressive responses than retired patients (Taylor and Saint-Cyr, 1990). The greatest complication in understanding the occurrence of depression in PD is the complex nature of depression itself. While many studies support the hypothesis that impaired serotonergic activity plays a role in depression, the number of serotonin receptors, transporters, and the efficacy of serotonin receptor-mediated signal transduction may be as important as the level of serotonin (Mann, 1999; Best et al., 2011). Numerous other neurotransmitter systems including noradrenaline and dopamine also play significant roles (Remy et al., 2005; Frisina et al., 2009; Miguelez et al., 2011). Further, significant deterioration of the serotonergic and noradrenergic systems may occur with PD (Jellinger, 1991; Halliday and McCann, 2010), often earlier than SNc degeneration (Braak et al., 2003). In this regard, it is interesting to note that some researchers report that depression may be an early symptom of the disease (Lemke, 2008; Poewe, 2008). Finally, we note that, given that LD is a precursor to both DA and noradrenaline, it is possible that the positive effects of LD on levels of these neurotransmitters may oppose the potentially depressive effects of lowered 5HT; while mood fluctuations are often reported in LD-treated PD patients (Black et al., 2005; Kulisevsky et al., 2007), links between LD therapy and depression remain equivocal (Mayeux et al., 1984; Taylor and Saint-Cyr, 1990; Choi et al., 2000; Pålhagen et al., 2008).

#### 3.1.4 Effects on the firing rate of DRN cells

As mentioned above, when the 5HT neurons in the dorsal raphe nucleus fire they release 5HT from the cell body similarly to the release at the terminal. In addition 5HT leaks out from the cell body into the extracellular space around the cell body and this accounts for about 30% of the release. The released 5HT binds to 5HT1a autoreceptors which lower firing rate when extracellular 5HT is high and raise firing rate when extracellular 5HT is low. For a review, see Adell et al. (2002). All three of these effects are in the model.

**Figure 5:**
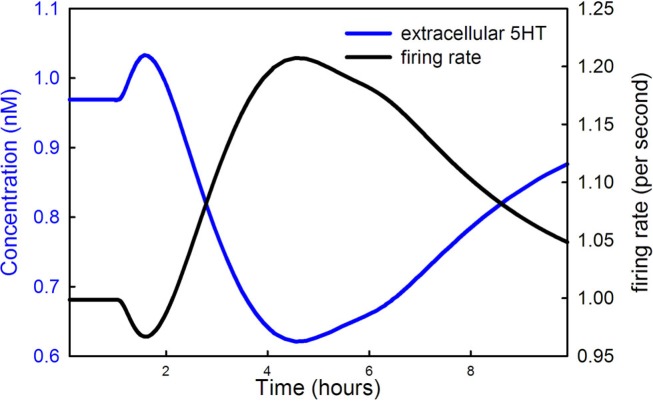
**LD changes the firing rate of DRN cells**. The 5HT concentration in the striatum (blue curve) and the firing rate of DRN neurons (black curve) are shown during an LD dose. The scale for the blue curve is on the left and the scale for the black curve is on the right.

Figure 5 shows the extracellular 5HT concentration (blue curve) and the firing rate of the DRN 5HT neuron as a function of time during the LD dose. Note that the scale for concentration is at the left and the scale for firing rate is at the right. In our model the normal firing rate is 1 spike/s consistent with much experimental evidence (Gartside et al., 1995; Feldman et al., 1997). During the main part of the LD dose, the firing rate goes up because extracellular 5HT goes down, a result of less 5HT being released from vesicles as described in Section 3.1.3. The lower binding to the 5HT1a autoreceptors causes firing rate to go up. But, what is the reason for the short drop in firing rate near the beginning of the LD dose? Recall that backflow from the vesicles causes cytosolic 5HT to increase during the initial part of the dose (the green curve in Figure 4A). The increase of cytosolic 5HT causes more 5HT to leak out into the extracellular space (the initial rise in the blue curve in Figure 5), which lowers the firing rate via the autoreceptors in the initial phase of the dose.

Finally we note that the increase in firing rate of DRN cells during the LD dose partially compensates for the drop in vesicular 5HT caused by competition with LD. If the firing rate did not go up, the drop in striatal extracellular 5HT would be larger than the 45% reported above.

### 3.2 Passive stabilization of extracellular DA

**Figure 6:**
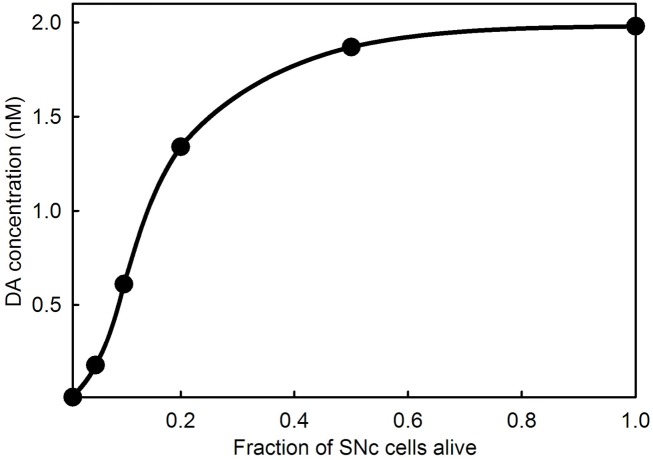
**Extracellular DA concentration as a function of the fraction of SNc cells alive**. The curve shows that the extracellular DA concentration in the striatum does not fall appreciably until 80% or more of the SNc cells have died.

An interesting and important feature of PD is that symptoms do not appear until a very large percentage (60–90%) of the cells in the SNc have died. Experiments with animal models (Abercrombie et al., 1990; Bezard et al., 2001; Dentresangle et al., 2001; Bergstrom and Garris, 2003) have shown that the DA content of striatal tissue declines proportionally to cell death but that the extracellular concentration of DA in the striatum remains near normal until 80% or so of the SNc cells have died. There have been many proposals to explain the homeostasis of extracellular DA in the face of SNc death. The simplest explanation was given by Garris and co-workers (Garris and Wightman, 1994; Garris et al., 1997; Bergstrom and Garris, 2003): as SNc cells die there is proportionally less DA released into the extracellular space, but the reuptake rate decreases proportionally because there are fewer DATs, and therefore the concentration of extracellular DA should remain the same. Garris refers to this phenomenon as “passive stabilization” to contrast it to other proposed mechanisms that require active adaptation. We verified the Garris mechanism by model calculations in Reed et al. (2009) and explained why extracellular DA does descend to zero when the fraction of cells remaining alive gets close to zero. As the DA terminals become more sparse, the probability of a DA molecule that has escaped from a synaptic cleft being reabsorbed by another DA terminal gets smaller and smaller. Figure 6 shows the passive stabilization of extracellular DA in the full model used in this paper. Passive stabilization explains why PD symptoms don’t appear until more than 80% of SNc cells have died. In Section 3.3, we study the effect of LD therapy when the fraction, *f*, of SNc cells remaining alive is in the range 0.01–0.2.

### 3.3 Dependence of the therapeutic time window on SNc cell death

The rationale behind LD treatment in Parkinson’s disease is that by supplying the precursor to DA the remaining SNc terminals in the striatum will store and release more DA. Typically, in the early stages of the disease, levodopa treatment is very efficacious in reducing symptoms for many hours. However, as the disease progresses the duration of benefit of an LD dose tends to become shorter and shorter until it approximates the half-life of LD in the serum (Marsden, 1980;Chase et al., 1987, 1989; Fabbrini et al., 1988; Mouradian et al., 1988; Papa et al., 1994). It has long been postulated that the “therapeutic time window” shortens because as more and more cells in the SNc die, there is less and less capacity in terminals to store DA in the striatum (Spencer and Wooten, 1984; Nutt, 1987). Experiments on rats have confirmed that the response to an LD dose changes with the degree of the denervation of the projection from the SNc to the striatum (Abercrombie et al., 1990).

**Figure 7:**
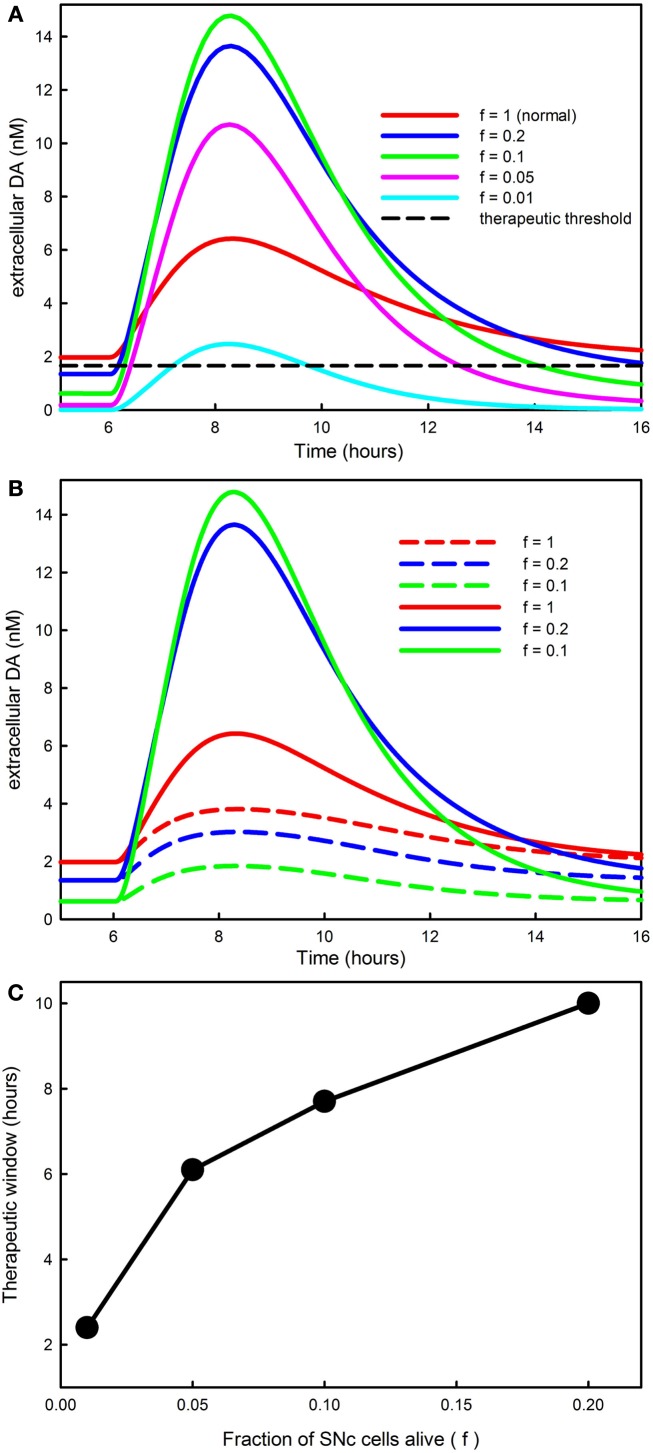
**The progression of PD affects DA in the striatum and the therapeutic time window**. **(A)** Shows the time course of extracellular DA in the striatum for different values of *f*, the fraction of SNc cells left alive. As *f* declines the curves get higher because there are fewer DATs to take up the DA released by 5HT neurons. However, when *f* is very small the peaks decline because removal mechanisms such as catabolism and diffusion become more important. The dashed black horizontal line in **(A)** represents the level of extracellular DA needed in the striatum for anti-Parkinsonian effects; see the Discussion in the text. **(B)** Reproduces the first three curves from [**(A)**; solid curves, *f* = 1, *f* = 0.2, *f* = 0.1]. The dashed curves in the same color show the amount of extracellular DA in the striatum that comes from the DA neurons. For a normal individual (*f* = 1) the DA neurons contribute approximately 60%, but as SNc cells die (*f* = 0.2, *f* = 0.1) most of the DA comes from the 5HT neurons. **(C)** Shows that the amount of time that extracellular DA stays above the therapeutic level [the dashed black line in **(A)**] declines as PD progresses until it becomes approximately 2 h.

In order to investigate how the fraction of SNc cells left alive, *f*, affects the time course of DA in the striatum, we ran simulations with *f* = 1 (normal), and *f* = 0.2, 0.1, 0.05, and 0.01 and the same LD dose as shown in Figure 2A. The results are shown in Figure 7A. The red curve shows the time course of the response for a normal individual. Ponzio et al. (1983) saw an increase by a factor of 2.5 in normal rats. Lindgren et al. (2010) and Abercrombie et al. (1990) found in normal rats that extracellular DA a little more than doubles during an LD dose. With our dose, extracellular DA goes up somewhat less than threefold in a normal individual (*f* = 1). The blue curve, corresponding to *f* = 0.2 (an 80% denervation), goes much higher than the red curve. The reason is that there are many fewer DATs to take up the DA that is released from the 5HT neurons. And, for *f* = 0.1, the green curve, the peak of the extracellular DA is still higher. However, for *f* = 0.05, the magenta curve, the peak gets lower, and for *f* = 0.01, the cyan curve, the time course of extracellular DA after an LD dose is quite low, even below the “normal” red curve.

The reason that the peaks come down when denervation is almost complete is that much of the released DA leaves the system. In our model, DA is lost from the extracellular space by three mechanisms: (1) it is transported back into the cytosol of DA terminals by the DATs; (2) it is catabolized to form homovanillic acid; (3) it is “removed” the system, which represents uptake by glial cells, uptake by blood vessels, or simple diffusion out of the striatum. In the normal striatum there is a dense innervation by terminals of SNc neurons, so a DA molecule that escapes from the synaptic cleft of a particular terminal is very likely to be taken up by the DATs on a different terminal. However, as the DA terminals become more sparse, the remaining terminals will be much farther apart, and a given molecule will be much more likely to be removed by one of the mechanisms in (3). In the model, removal rate is proportional to the current extracellular DA concentration with constant of proportionality *k_rem_* and this constant depends on *f* the fraction of SNc cells (i.e., the fraction of DA terminals) left alive. The correct functional form of this dependence is a difficult question. For the simulations here, we take *k_rem_* = *k_o_*/*f*.

Thus there are two competing effects. As *f* gets smaller, the smaller number of DATs tends to increase the height of the peak, but the greater removal from the system tends to lower the peak. The first effect dominates when *f* is relatively large, i.e., in the range 0.1–1, and the second effect dominates when *f* is very small, i.e., in the range 0.01–0.05.

The curves in Figure 7A show the time course of total extracellular DA in the striatum during an LD dose. But how much of this extracellular DA comes from DA neurons and how much from 5HT neurons? The answers can be seen in Figure 7B where the dashed curves show the contribution of the DA neurons in the cases *f* = 1, 0.2, 0.1; for comparison we have regraphed the total DA curves from Figure 7A. For a normal individual, the DA neurons contribute about 60% of the DA in the striatum during the dose. However, when most of the SNc cells have died (*f* = 0.2, 0.1), the vast majority of the increase of DA in the striatum comes from 5HT neurons. This is consistent with animal experiments. Tanaka et al. (1999) and Lindgren et al. (2010) showed that lesioning of the 5HT projection to the striatum decreases DA substantially in the striatum during an LD dose and Carta et al. (2007) and Lindgren et al. (2010) showed that 5HT autoreceptor agonists decrease the dyskinesias caused by high pulses of DA in the striatum.

When clinicians and experimentalists refer to the “therapeutic time window” of an LD dose they mean the amount of time after the dose that Parkinsonian symptoms are suppressed. Our model does not include downstream electrophysiological behavior in the basal ganglia, nor does it include the resulting motor behavior. Nevertheless, we can make a reasonable assumption about how to calculate the therapeutic time window in the model. In the lower left corner of Figure 7A, the flat curves represent the steady state levels of extracellular DA in the striatum for the different choices of *f*. The therapeutic level of extracellular DA necessary to prevent symptoms must be below the red line (since the normal patient doesn’t have symptoms) and above the blue line (since the *f* = 0.2 patient does have symptoms). We’ll take the therapeutic level of extracellular DA necessary to prevent symptoms as halfway between the red and blue line, the dashed black line in Figure 7A. For each choice of *f*, the therapeutic time window is then the amount of time that the extracellular DA curve stays above the dashed line.

Figure 7C shows the therapeutic time window in the model as a function of *f*, the fraction of SNc cells left alive. The therapeutic time window shortens from about 10 h when *f* = 0.2 to a little more than 2 h when *f* = 0.01, close to the half-life of the LD dose in the blood. This is consistent with many clinical and experimental observations; see for example Fabbrini et al. (1988) and Olanow et al. (2006). The shortening of the period of efficacy of the LD dose is caused both by the reduced capacity of the remaining SNc to store DA released by 5HT neurons and by the loss of DA from the system as DA terminals become more sparse.

#### 3.3.1 Pulsatile DA in the striatum and dyskinesias

There is a large amount of clinical and experimental evidence that large pulses of extracellular DA in the striatum are associated with dyskinesias. For example, PD patients with dyskinesias have higher levels of synaptic DA after an LD dose (de la Fuente-Fernandez et al., 2004). Rats with lesioned SNc projections have higher extracellular DA levels in the striatum if they have dyskinesias (Lindgren et al., 2010). And, DA agonists themselves can cause dyskinesias (Rascol et al., 2001). Continuous i.v. infusions of LD cause few dyskinesias (Mouradian et al., 1987). As we have explained above (Figure 7A), the primary cause of these high pulses is DA release by 5HT neurons in the striatum.

We also explained that the height of the pulses increases as PD progresses and the fraction, *f*, of SNc cells alive declines from 0.2 to 0.05. This is consistent with the results of Mouradian et al. (1988) who showed that the dose of LD necessary to cause dyskinesias declines as PD progresses. They attribute the higher pulse to the declining ability of the remaining SNc neurons to store the DA produced from LD (the “storage hypothesis”). Our model calculations suggest that the pulse gets higher as PD progresses because fewer and fewer DATs are available to take up the DA released by 5HT neurons.

However, it is also clear that this is not the whole story. Nutt et al. (2000) showed that repeated doses of LD are necessary to produce dyskinesias in rats and denervation is not necessary. Other studies showed that the response to DA agonists is different in PD patients on LD compared to LD-naive PD patients (Bravi et al., 1994; Verhagen Metman et al., 1997), suggesting that LD may cause post-synaptic changes. Since then a whole host of results, reviewed in Cenci and Lundblad (2006) and Cenci (2007), have shown that chronic LD dosing is associated with altered intracellular trafficking of DA and glutamate receptors in striatal cells (Hallett et al., 2005; Guigoni et al., 2007), altered expression and regulation of transcription factors in striatal cells (Aubert et al., 2007), and altered oscillatory synchronization frequencies in the Subthalamic nucleus and the Globus Pallidus (Foffani et al., 2005; Alonso-Frech et al., 2006). These downstream changes induced by high pulses of extracellular DA in the striatum are not part of our current model.

### 3.4 The effects of 5HT1a and 5HT1b agonists

The 5HT1a autoreceptors on 5HT cell bodies in the DRN regulate 5HT neuron firing rate, depressing it when extracellular 5HT rises and raising it when extracellular 5HT falls (Adell et al., 2002). The 5HT1b autoreceptors on 5HT terminals in the striatum downregulate 5HT release and synthesis when extracellular 5HT rises and increase synthesis and release when extracellular 5HT falls (Adell et al., 2002). A number of recent experimental studies have explored the effects of giving 5HT1a and/or 5HT1b agonists in combination with LD in animal models (Bibbiani et al., 2001; Jackson et al., 2004;Carta et al., 2007, 2008; Lindgren et al., 2010). Here we study the effects of 5HT1a and 5HT1b agonists using our mathematical model.

In our model, the agonists affect the autoreceptors in the same way that increasing the level of 5HT would affect the autoreceptors. Our dose units for the 5HT1a and 5HT1b agonists are multiples of the normal extracellular 5HT concentration in the Raphe and the striatum, respectively. Thus a dose of 3 units of a 5HT1a agonist acts in the same way as raising extracellular 5HT from normal to 4 times normal. We will conduct our experiments on a model patient or animal with *f* = 0.1; thus 90% of the projections from the SNc to the striatum have been removed.

**Figure 8:**
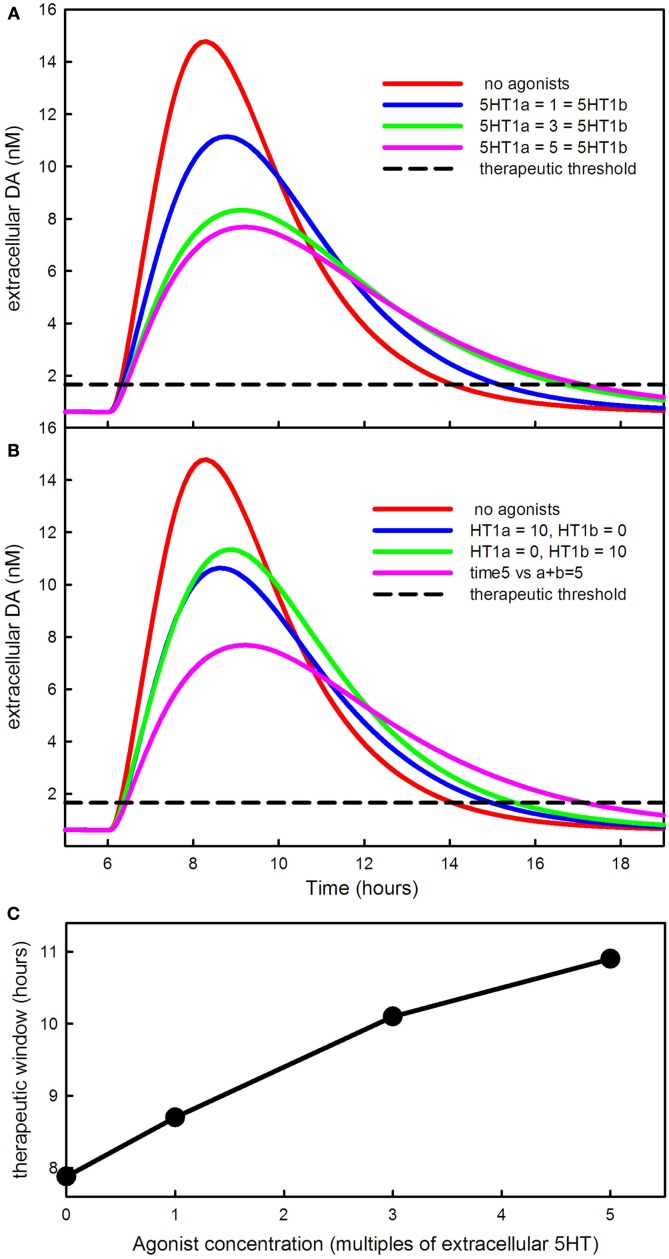
**The effects of 5HT agonists on extracellular DA in the striatum**. **(A)** Shows the time course of extracellular DA in the striatum in the presence of larger and larger equal doses of 5HT1a and 5HT1b agonists. The agonists lower the peak and stretch out the tail of the DA concentration. **(B)** Shows that the DA peak is lowered much more by dividing the dose between the agonists instead of giving either agonist alone. **(C)** Shows how much the therapeutic time window is increased by the agonists.

Figure 8A shows the effect of increasing doses of a 5HT1a agonist and a 5HT1b agonist given together. The higher the agonist concentration the lower the DA pulse in the striatum after an LD dose, and the longer the time period over which the LD dose has an effect. The curves are similar to the curves in Lindgren et al. (2010), Figure 4A. One can see that the effect of the agonists saturates because the response to 5 units of each agonist (the magenta curve) is not much different from the response to 3 units. This happens because, in the model, the influence of the autoreceptors on firing rate, synthesis, and release saturates. Figure 8B shows the synergistic effect of the two types of agonists observed in Carta et al. (2007, 2008). Ten units of agonist divided equally between the two types of agonists lowers the DA pulse much more than 10 units of a single agonist.

The dashed curve in Figures 8A,B is the therapeutic threshold discussed in Section 3.3. The agonists increase the time above the therapeutic threshold (C). This is because, in the presence of the agonists, the 5HT neuron fires more slowly and also releases less neurotransmitter per action potential. As a result, the DA that has been synthesized in the 5HT neuron from LD is released from the terminal in the striatum more slowly. Bibbiani et al. (2001) showed that the 5HT1a agonist sarizotan increases the time above the therapeutic threshold in rats. Thus, giving 5HT1a and 5HT1b agonists has two therapeutic benefits: (1) the DA pulse in the striatum is lower and is therefore less likely to cause dyskinesias as discussed above; (2) the therapeutic time window becomes longer (Figure 8C).

## 4 Discussion

We have used our mathematical model to investigate some biochemical and electrophysiological consequences of dosing with LD. It has been known for a long time that LD competes with tyrosine and tryptophan and the other large neutral amino acids at the BBB; it has even been recommended that LD patients consume low protein meals (Pincus and Barry, 1987; Khor and Hsu, 2007). Less well understood are the facts that tyrosine, tryptophan, and LD are taken up by all cells and that LD in 5HT neurons competes with 5HTP for AADC and that DA competes with 5HT for VMAT. We explained in Section 3.1 of Results why LD and DA outcompete 5HTP and 5HT at these two steps, thus essentially turning 5HT neurons into DA neurons during the dose. In Section 3.2 we explained the mechanism of passive stabilization of extracellular DA in the striatum that explains why Parkinsonian symptoms do not appear until approximately 80% of the cells in the SNc have died. This mechanism, first proposed by Garris, Wightman, and co-workers (Wightman and Zimmerman, 1990; Bergstrom and Garris, 2003) and verified in our model (Reed et al., 2009) is not well known in the community.

In Section 3.3 we investigated how the time course of extracellular DA in the striatum changes during an LD dose as PD progresses and more cells in the SNc die. We saw that at first the DA peaks get higher and narrower as more SNc cells die. This is because there are fewer and fewer DATs available to take up the DA released from 5HT neurons. However, when very few SNc cells are left (below 5%) the peak of the extracellular DA curve comes back down because the lack of DA terminals makes it less and less likely that a DA molecule that escapes from a synapse will be taken up by another terminal before being metabolized or diffusing out of the system. In our model, the therapeutic time window in which the LD dose relieves PD symptoms gradually shortens to 2 h as PD progresses and more and more SNc cells die. There may very well be other mechanism that contribute to shortening (see below), but we have shown that the biochemistry of LD in 5HT neurons and the interplay between release from 5HT terminals and uptake by DA terminals explains the phenomenon.

In Section 3.4 we used the model to investigate the effects of 5HT1a and 5HT1b agonists. As many experimentalists have found, the agonists lower the height of the extracellular DA peak in the striatum (Carta et al., 2007, 2008) and increase the therapeutic time window of the LD dose (Bibbiani et al., 2001). We also see the synergistic effect of 5HT1a and 5HT1b agonists given together (Carta et al., 2007). The underlying reason for the synergy is that the effects of the agonists are governed by binding to the autoreceptors. The binding is not a linear function of concentration but saturates because there are only finitely many receptors. Thus one gets more inhibition of DA release from a moderate amount of both agonists than one gets from a large amount of either.

The problem of dyskinesias induced by LD therapy is extremely important clinically but very complicated scientifically. Between the LD dose and the dyskinetic behavior there are four levels of mechanisms. The first level is the absorption of LD from the gut, the metabolism in the blood, and the transport across the BBB. The second level is the biochemistry of LD in 5HT neurons and the interaction between the DA released from 5HT terminals in the striatum with the remaining SNc terminals. The third level encompasses the cellular and gene expression changes in striatal cells caused by the high pulses of DA released from 5HT neurons. The fourth level consists of the electrophysiological changes that are thereby induced in the downstream nuclei of the basal ganglia. Our model addresses questions only at the first and second level. There is lots of biological information but no mathematical modeling yet at the third level. There is a considerable body of mathematical work that tries to explain and characterize the dynamical firing patterns in the downstream nuclei of the basal ganglia as PD progresses, both before and after deep brain stimulation of the subthalamic nucleus (Terman et al., 2002; Best et al., 2007; van Albada and Robinson, 2009; Hahn and McIntyre, 2010; Park et al., 2011).

Even on the first and second levels, which are covered by our model, there are many biological details that we have not included. Our model does not include a cell body for the SNc neuron. Lindgren et al. (2010) have shown that LD increases extracellular DA in the SNc and this will presumably lower the firing rate of SNc cells and therefore lower release in the striatum. This was not included because our main focus in this paper is on the effects of LD on 5HT neurons. Many other known phenomena where not included because we wanted to keep the model as simple as possible we felt that their effects would be relatively small and would not affect the main conclusions of this paper. For example, the SNc sends excitatory projections to the DRN and the DRN sends inhibitory projections to the SNc (Di Matteo et al., 2008; Guiard et al., 2008). Lesioning the SNc causes hyperinnervation of the striatum by 5HT neurons (Maeda et al., 2003). And, finally, the 5HT neurons in the RN send ascending projections to a large number of different brain regions including medial prefrontal cortex (mPFC), motor cortex, hypothalamus, hippocampus, amygdala, and the basal ganglia. And, some of these brain regions, substantia nigra, amygdala, mPFC, and hypothalamus send projections back to the RN (Monti, 2010). As we have explained, all of these circuits will be affected by LD doses and thus may influence the firing rates of RN neurons and therefore the release of DA in the striatum.

It is known that SERTs can transport DA and that DATs can transport 5HT, so it is natural to wonder how these phenomena would affect the basic findings and conclusions in this paper. DATs transport significant amounts of 5HT only in SERT-deficient mice where the extracellular concentration of 5HT is very high (Fox et al., 2008). During an LD dose, the 5HT concentration in the striatum goes down significantly below normal, so it is very unlikely that 5HT uptake by DATs would play any role. However, SERTs can transport significant amounts of DA at concentrations that are in the same range as the DA concentrations seen in the striatum during LD doses (Larsen et al., 2011). To test how large this effect is we added DA transport into 5HT terminals by SERTs to our model using a *K_m_* 100 times larger for DA than for 5HT and a *V_max_* 4.5 times higher for DA as found by Larsen et al. (2011). For a normal individual (*f* = 1), the addition of this transport caused almost no change in the time course of extracellular DA in the striatum (simulation not shown). This is consistent with the results of Larsen et al. (2011) who found that blockade of the SERTs increases the *in vivo* clearance time of DA in the hippocampus but not in the striatum. For an individual with advanced PD (*f* = 0.1), the addition of this transport caused a modest (13%) decrease in the peak value of DA in the striatum during the LD dose (simulation not shown). Thus, the addition of DA transport by SERTs to the model causes small changes in some of the curves in Figures 7A,B, but does not affect the basics findings and conclusions of the paper.

Our model makes predictions about the firing rates of RN cells that can be checked experimentally. First of all, we predict that during most of the LD dose the firing rates of RN cells will go up by approximately 20% (Figure 5). However, we also predict that at the onset of the dose, the firing rate will will dip down. In the model this is because the competition of DA and 5HT for VMAT causes a backflow of 5HT from the vesicles of into the cytosol. The higher cytosolic 5HT concentrations at the beginning of the dose cause more leakage of 5HT to the extracellular space where it inhibits firing via the 5ht1a autoreceptors. Experimental confirmation would provide strong support for the backflow out of the vesicles proposed in Floor et al. (1995) and Wallace (2007).

The purpose of mathematical models is to investigate the causal relationships between phenomena measured by experimentalists and seen by clinicians, thereby increasing the understanding of complex biological systems. To be useful, such models must be based on real physiology and the creation of such models is not easy. However, if one has a model that represents (part of) the underlying physiology well, then *in silico* experiments are quick and inexpensive. The model provides a quantitative way of thinking about the phenomena being investigated and may suggest new hypotheses that can be checked by animal experiments. Thus, modeling is a different tool, which, when combined with animal experiments and clinical trials, can shed some light on the complicated pharmacological, electrophysiological, and behavioral issues in PD and LD dosing.

## Conflict of Interest Statement

The authors declare that the research was conducted in the absence of any commercial or financial relationships that could be construed as a potential conflict of interest.

## Figures and Tables

**Figure A1:**
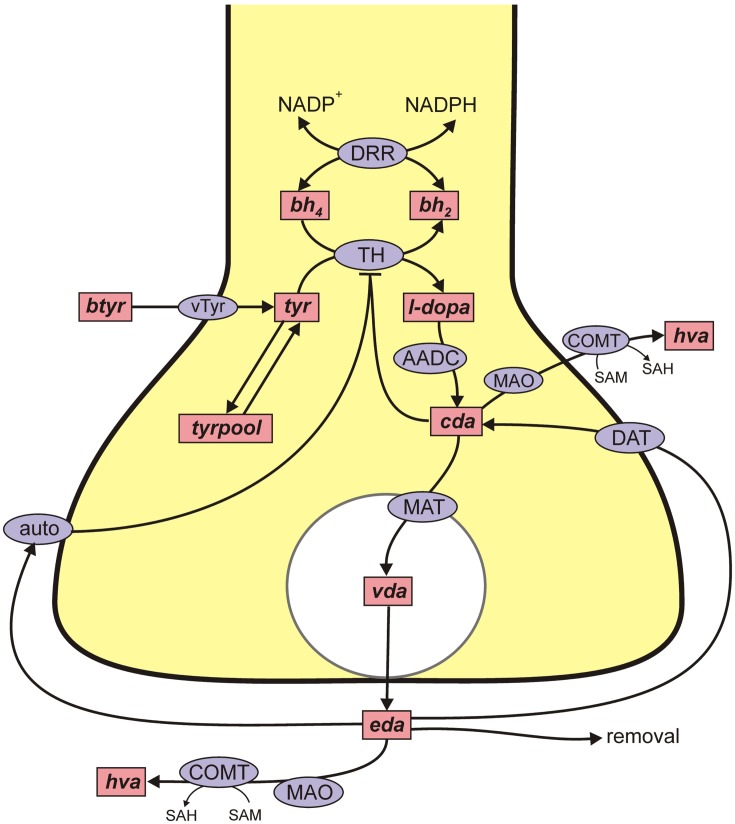
**DA metabolism in a DA terminal**. Rectangular boxes indicate substrates (see Table A1) and blue ellipses contain the acronyms of enzymes or transporters: *V*_Ltyr_, l-amino acid transporter; DRR, dihydrobiopterin reductase; TPH, tyrosine hydroxylase; AADC, aromatic amino acid decarboxylase; MAT, vesicular monoamine transporter; DAT, DA reuptake transporter; auto, DA autoreceptors; MAO, monoamine oxidase; COMT, catacholamine methyltransferase. Removal means uptake by capillaries or glial cells or diffusion out of the system. In Table A1 various shorter names are used for the dopamine variables in the dopamine terminal and the variables all start with “d” to distinguish them from the dopamine variables in the 5HT terminal and cell body.

**Figure A2:**
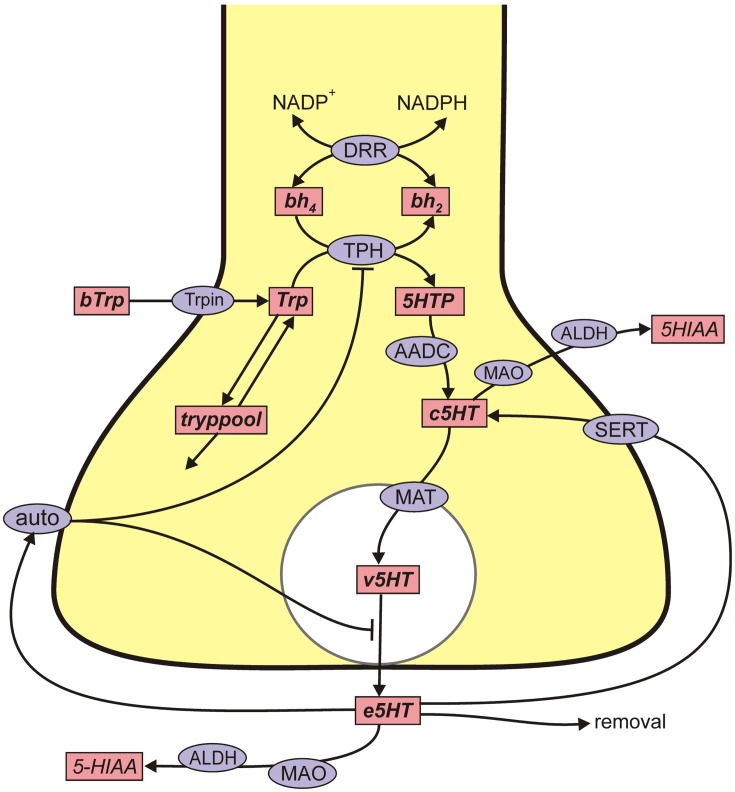
**5HT metabolism in a 5HT terminal**. Rectangular boxes indicate substrates (see Table A1) and blue ellipses contain the acronyms of enzymes or transporters: *V*_Ltryp_, l-amino acid transporter; DRR, dihydrobiopterin reductase; TPH, tryptophan hydroxylase; AADC, aromatic amino acid decarboxylase; MAT, vesicular monoamine transporter; SERT, 5HT reuptake transporter; auto, 5HT autoreceptors; MAO, monoamine oxidase; ALDH, aldehyde dehydrogenase. Removal means uptake by capillaries or glial cells or diffusion out of the system. In Table A1 various shorter names are used for the 5HT variables in the 5HT terminal and 5HT cell body. The cell body names all start with “r.”

## Appendix

A schematic diagram of the most important variables in the mathematical model is given in Figure 1. More detailed diagrams of dopamine and serotonin metabolism and full names of the enzymes are given in Figure A1 (page 3) and Figure A2 (page 4). Table A1 lists all the variables and their steady state concentrations (page 5). The full set of differential equations are given next (page 6), followed by a discussion of modeling issues (page 8). Tables A2– A5 give all parameter values in the model (page 12).

**Table A1:** **Variables**.

Abbreviation	Steady state (μM)	Full name
**5HT TERMINAL**		
bh2	0.14	Dihydrobiopterin
bh4	0.86	Tetrahydrobiopterin
Tryp	20.1	Tryptophan
Htp	2.23	5-Hydroxytryptophan
Cht	0.50	Cytosolic 5HT
Vht	20.4	Vesicular 5HT
Eht	0.00073	Extracellular 5HT
Hia	5.21	5-Hydroxyindoleacetic acid
Tryppool	143	The tryptophan pool
**5HT CELL BODY**		
rbh2	0.14	Dihydrobiopterin
rbh4	0.86	Tetrahydrobiopterin
Rtryp	20.1	Tryptophan
Rhtp	2.23	5-Hydroxytryptophan
Rcht	0.49	Cytosolic 5HT
Rvht	20.26	Vesicular 5HT
Reht	0.00097	Extracellular 5HT
Rhia	5.1	5-Hydroxyindoleacetic acid
Rtryppool	143	The tryptophan pool
**DA TERMINAL**		
dbh2	0.69	Dihydrobiopterin
dbh4	0.31	Tetrahydrobiopterin
Dtyr	126.9	Tyrosine
Dld	0.36	3,4-Dihyroxyphenylalanine (l-DOPA)
Dcda	2.65	Cytosolic dopamine
Dvda	79.1	Vesicular dopamine
Deda	0.00198	Extracellular dopamine
Dhva	7.68	Homovanillic acid
Dtyrpool	952	The tyrosine pool
**OTHER**		
Sld	–	Serum levodopa
Styr	63	Serum tyrosine
Stryp	82	Serum tryptophan
5ld	–	5HT terminal LD
5cda	–	5HT terminal cytosolic DA
5vda	–	5HT terminal vesicular DA
Rld	–	5HT cell body LD
Rcda	–	5HT cell body cytosolic DA
Rvda	–	5HT cell body vesicular DA

**Table A2:** **Kinetic parameters (μM, μM/h, /h): 5HT terminal**.

Velocity	Parameter	Model value	Literature value	Reference
*V*_AADChtp_	Aromatic amino acid decarboxylase			
	$K_{m}^{5htp}$	160	160	Sumi et al. (1990)
	$K_{m}^{5ld}$	130	130	Siaterli et al. (2003)
	*V_max_*	400		*
*V_DRR_*	Dihydropteridine reductase			
	$K_{m}^{bh2}$	100	4–754	Armarego et al. (1986), Bailey and Ayling (1983)
	$K_{m}^{\text{NADPH}}$	75	29–770	Firgaira et al. (1981, 1987), Schomburg and Schomburg (2005)
	$V_{max}^{f}$	5000		*
	$K_{m}^{bh4}$	10	1.1–17	Craine et al. (1972), Firgaira et al. (1981)
	$K_{m}^{\text{NADP}}$	75	29–770	Firgaira et al. (1981, 1987), Schomburg and Schomburg (2005)
	$V_{max}^{b}$	3		*
*V*_Lstryp_	Neutral amino acid transporter			
	$K_{m}^{stryp}$	15	15	Kilberg and Haussinger (1992)
	$K_{m}^{styr}$	64	64	Kilberg and Haussinger (1992)
	$K_{m}^{sld}$	32	28–102	Hu and Li (2011), Sampaio-Maia et al. (2001)
	*V_max_*	214		*
*V*_MATcht_	Vesicular monoamine transporter			
	$K_{m}^{cht}$	0.2	0.123–0.253	Slotkin et al. (1978), Rau et al. (2006)
	$K_{m}^{5cda}$	3	0.2–10	Sherman and Henry (1983), Near (1986), Volz et al. (2006)
	*V_max_*	40		*
	*k_out_*	0.4		*
*V*_SERT_	Serotonin transporter			
	*K_m_*	0.17	0.05–1	Feldman et al. (1997), Bunin et al. (1998), Daws et al. (2005)
	*V_max_*	4700		*
*V*_TPH_	Tryptophan hydroxylase			
	*K_trp_*	40	40	McKinney et al. (2005)
	*K_bh4_*	20	20	McKinney et al. (2005)
	*V_max_*	400		*
	*K_i_* (substrate inhibition)	1000	970	McKinney et al. (2005)
**CATABOLISM AND DIFFUSION**				
$V_{\text{cht}}^{catab}$	*K_m_*	95	94–95	Fowler and Ross (1984), Gottowik et al. (1993)
	*V_max_*	1000		*
$V_{\text{eht}}^{catab}$	*K_m_*	95	94–95	Fowler and Ross (1984), Gottowik et al. (1993)
	*V_max_*	1000		*
$V_{\text{tryp}}^{catab}$	*K_m_*	20		*
	*V_max_*	74		*
$k_{\text{tryppool}}^{catab}$		0.8		*
$k_{\text{hia}}^{catab}$		1	1	Echizen and Freed (1984)
*k_rem_*		400		*

**See text*.

**Table A3:** **Kinetic parameters (μM, μM/h, /h): 5HT cell body**.

Velocity	Parameter	Model value	Literature value	Reference
*V*_AADCrhtp_	Aromatic amino acid decarboxylase			
	$K_{m}^{rhtp}$	160	160	Sumi et al. (1990)
	$K_{m}^{rld}$	130	130	Siaterli et al. (2003)
	*V_max_*	400		*
*V*_DRR_	Dihydropteridine reductase			
	$K_{m}^{rbh2}$	100	4–754	Armarego et al. (1986), Bailey and Ayling (1983)
	$K_{m}^{\text{NADPH}}$	75	29–770	Firgaira et al. (1981, 1987), Schomburg and Schomburg (2005)
	$V_{max}^{f}$	5000		*
	$K_{m}^{rbh4}$	10	1.1–17	Craine et al. (1972), Firgaira et al. (1981)
	$K_{m}^{\text{NADP}}$	75	29–770	Firgaira et al. (1981, 1987), Schomburg and Schomburg (2005)
	$V_{max}^{b}$	3		*
*V*_MATrcht_	Vesicular monoamine transporter			
	$K_{m}^{rcht}$	0.2	0.123–0.253	Slotkin et al. (1978), Rau et al. (2006)
	$K_{m}^{rcda}$	3	0.2–10	Sherman and Henry (1983), Near (1986), Volz et al. (2006)
	$V_{max}$	40		*
	*k_out_*	0.4		*
*V*_SERT_	Serotonin transporter			
	*K_m_*	0.17	0.05–1	Feldman et al. (1997), Bunin et al. (1998), Daws et al. (2005)
	*V_max_*	4700		*
*V*_TPH_	Tryptophan hydroxylase			
	*K_rtrp_*	40	40	McKinney et al. (2005)
	*K_rbh4_*	20	20	McKinney et al. (2005)
	*V_max_*	400		*
	*K_i_* (substrate inhibition)	1000	970	McKinney et al. (2005)
*V*_Lstryp_	Neutral amino acid transporter			
	$K_{m}^{stryp}$	15	15	Kilberg and Haussinger (1992)
	$K_{m}^{styr}$	64	64	Kilberg and Haussinger (1992)
	$K_{m}^{sld}$	32	28–102	Hu and Li (2011), Sampaio-Maia et al. (2001)
	*V_max_*	214		*
**CATABOLISM AND DIFFUSION**				
$V_{\text{rcht}}^{catab}$	*K_m_*	95	94–95	Fowler and Ross (1984), Gottowik et al. (1993)
	*V_max_*	1000		*
$V_{\text{reht}}^{catab}$	*K_m_*	95	94–95	Fowler and Ross (1984), Gottowik et al. (1993)
	*V_max_*	1000		*
$V_{\text{rtryp}}^{catab}$	*K_m_*	20		*
	*V_max_*	74		*
$k_{\text{rtryppool}}^{catab}$		0.8		*
$k_{\text{rhia}}^{catab}$		1	1	Echizen and Freed (1984)
*k_rem_*		400		*
*k_leak_*		14		

**See text*.

**Table A4:** **Kinetic parameters (μM, μM/h, /h): DA terminal**.

Velocity	Parameter	Model value	Literature value	Reference
*V*_AADC_	Aromatic amino acid decarboxylase			
	*K_m_*	130	130	Siaterli et al. (2003)
	*V_max_*	10,000		*
*V*_DAT_	Dopamine transporter			
	*K_m_*	0.2	0.2–2	Jones et al. (1995), Schmitz et al. (2003)
	*V_max_*	8000		*
*V*_DRR_	Dihydropteridine reductase			
	*K*_bh2_	100	4–754	Armarego et al. (1986), Bailey and Ayling (1983)
	*K*_NADPH_	75	29–770	Firgaira et al. (1981, 1987), Schomburg and Schomburg (2005)
	$V_{max}^{f}$	5000		*
	*K_bh4_*	10	1.1–17	Craine et al. (1972), Firgaira et al. (1981)
	*K*_NADP_	75	29–770	Firgaira et al. (1981, 1987), Schomburg and Schomburg (2005)
	$V_{max}^{b}$	80		*
*V*_MAT_	Vesicular monoamine transporter			
	*K_m_*	3	0.2–10	Sherman and Henry (1983), Near (1986), Volz et al. (2006)
	*V_max_*	236		*
	*k_out_*	0.4		*
*V*_TH_	Tyrosine hydroxylase			
	*K_tyr_*	46	46	Royo et al. (2004)
	*K_bh4_*	60	13	Royo et al. (2004)
	*V_max_*	3500		*
	*K_i_* (cda)	110	110	Morgenroth et al. (1976)
	*K_i_* (substrate inhibition)	160	160	Royo et al. (2004); 160 Nakashima et al. (1999)
*V*_Lstyr_	Neutral amino acid transporter			
	$K_{m}^{styr}$	64	64	Kilberg and Haussinger (1992)
	$K_{m}^{stryp}$	15	15	Kilberg and Haussinger (1992)
	$K_{m}^{sld}$	32	28–102	Hu and Li (2011), Sampaio-Maia et al. (2001)
	*V_max_*	1840		*
*tyr*↔ *Tyrpool*				
	*k*_1_	6		*
	*k*_−1_	0.6		*
**CATABOLISM AND DIFFUSION**				
$V_{\text{eda}}^{catab}$	*K_m_*	3	3.3	Rivett and Roth (1982)
	*V_max_*	30		*
$k_{\text{dtyr}}^{catab}$		0.2		*
$k_{\text{dcda}}^{catab}$		10		*
$k_{\text{dhva}}^{catab}$		3.45	3.45	Karoum et al. (1977), Dedek et al. (1979)
$k_{\text{dtyrpool}}^{catab}$		0.2		*
*k_rem_*		400		*

**See text*.

**Table A5:** **Kinetic parameters (μM, μM/h, /h): LD and DA in the 5HT neuron**.

Velocity	Parameter	Model value	Literature value	Reference
*V*_AADC5ld_	Aromatic amino acid decarboxylase			
	$K_{m}^{5ld}$	130	130	Siaterli et al. (2003)
	$K_{m}^{5htp}$	160	160	Sumi et al. (1990)
	*V_max_*	400		*
*V*_AADCrld_	Aromatic amino acid decarboxylase			
	$K_{m}^{rld}$	130	130	Siaterli et al. (2003)
	$K_{m}^{rhtp}$	160	160	Sumi et al. (1990)
	*V_max_*	400		*
*V*_Lsld_	Neutral amino acid transporter			
	$K_{m}^{sld}$	32	28–102	Hu and Li (2011), Sampaio-Maia et al. (2001)
	$K_{m}^{stryp}$	15	15	Kilberg and Haussinger (1992)
	$K_{m}^{styr}$	64	64	Kilberg and Haussinger (1992)
	*V_max_*	1750		*
*V*_MAT5cda_	Vesicular monoamine transporter			
	$K_{m}^{5cda}$	0.2	0.2–10	Sherman and Henry (1983), Near (1986), Volz et al. (2006)
	$K_{m}^{cht}$	3	0.123–0.253	Slotkin et al. (1978), Rau et al. (2006)
	*V_max_*	40		*
	*k_out_*	0.4		*
*V*_MATrcda_	Vesicular monoamine transporter			
	$K_{m}^{rcda}$	0.2	0.2–10	Sherman and Henry (1983), Near (1986); Volz et al. (2006)
	$K_{m}^{rcht}$	3	0.123–0.253	Slotkin et al. (1978), Rau et al. (2006)
	*V_max_*	196.8		*
	*k_out_*	0.4		*
Catabolism				
	$k_{5\text{cda}}^{catab}$	10		*
	$k_{\text{rcda}}^{catab}$	10		*

The 5HT terminal:

$$\begin{array}{l} \frac{d\left(bh2\right)}{dt}=V_{\text{TPH}}\left(tryp,bh4,eht\right)-V_{\text{DRR}}\left(bh2,\text{NADPH},bh4,\text{NADP}\right)\\ \frac{d\left(bh4\right)}{dt}=V_{\text{DRR}}\left(bh2,\text{NADPH},bh4,\text{NADP}\right)-V_{\text{TPH}}\left(tryp,bh4,eht\right)\\ \frac{d\left(tryp\right)}{dt}=V_{\text{Lstryp}}\left(stryp,styr,sld\right)-V_{\text{TPH}}\left(tryp,bh4,eht\right)\\ \;-\frac{400\cdot \left(tryp\right)}{20+tryp}+\left(0.6\right)\cdot \left(tryppool\right)-V_{\text{tryp}}^{catab}\left(tryp\right)\\ \frac{d\left(htp\right)}{dt}=V_{\text{TPH}}\left(tryp,bh4,eht\right)-V_{5\text{AADC}}\left(htp,5ld\right)\\ \frac{d\left(cht\right)}{dt}=V_{5\text{AADC}}\left(htp,5ld\right)-V_{5\text{MAT}}\left(cht,5cda,vht\right)+V_{\text{SERT}}\left(eht\right)-V_{\text{cht}}^{catab}\left(cht\right)\\ \frac{d\left(vht\right)}{dt}=V_{5\text{MAT}}\left(cht,5cda,vht\right)-\text{TautoR}\left(eth\right)\cdot \left(RFIRE\left(rinput\left(t\right),reht\right)\right)\cdot \left(vht\right)\\ \frac{d\left(eht\right)}{dt}=\text{TautoR}\left(eth\right)\cdot \left(RFIRE\left(rinput\left(t\right),reht\right)\right)\cdot \left(vht\right)-V_{\text{SERT}}\left(eht\right)\\ \;-V_{\text{eht}}^{catab}\left(eht\right)-k_{rem}\cdot \left(eht\right)\\ \frac{d\left(hia\right)}{dt}=V_{\text{cht}}^{catab}\left(cht\right)+V_{\text{eht}}^{catab}\left(eht\right)-k_{\text{hia}}^{catab}\cdot \left(hia\right)\\ \frac{d\left(tryppool\right)}{dt}=\frac{400\cdot \left(tryp\right)}{20+tryp}-\left(0.6\right)\cdot \left(tryppool\right)-k_{\text{tryppool}}^{catab}\cdot \left(tryppool\right) \end{array}$$

The 5HT cell body:

$$\begin{array}{l} \frac{d\left(rbh2\right)}{dt}=V_{\text{TPH}}\left(rtryp,rbh4,reht\right)-V_{\text{RDRR}}\left(rbh2,\text{NADPH},rbh4,\text{NADP}\right)\\ \frac{d\left(rbh4\right)}{dt}=V_{\text{RDRR}}\left(rbh2,\text{NADPH},rbh4,\text{NADP}\right)-V_{\text{RTPH}}\left(rtryp,rbh4,reht\right)\\ \frac{d\left(rtryp\right)}{dt}=V_{\text{Lstryp}}\left(stryp,styr,sld\right)-V_{\text{RTPH}}\left(rtryp,rbh4,reht\right)\\ \;-\frac{400\cdot \left(rtryp\right)}{20+rtryp}+\left(0.6\right)\cdot \left(rtryppool\right)-V_{\text{rtryp}}^{catab}\left(rtryp\right)\\ \frac{d\left(rhtp\right)}{dt}=V_{\text{TPH}}\left(rtryp,rbh4,reht\right)-V_{\text{RAADC}}\left(rhtp,rld\right)\\ \frac{d\left(rcht\right)}{dt}=V_{\text{RAADC}}\left(rhtp,rld\right)-V_{\text{RMAT}}\left(rcht,rcda,rvht\right)+V_{\text{SERT}}\left(reht\right)\\ \;-V_{\text{rcht}}^{catab}\left(rcht\right)-k_{leak}\cdot \left(rcht-reht\right)\\ \frac{d\left(rvht\right)}{dt}=V_{\text{RMAT}}\left(rcht,rcda,rvht\right)-\left(RFIRE\left(rinput\left(t\right),reht\right)\right)\cdot \left(rvht\right)\\ \frac{d\left(reht\right)}{dt}=\left(RFIRE\left(rinput\left(t\right),reht\right)\right)\cdot \left(rvht\right)-V_{\text{SERT}}\left(reht\right)-V_{\text{reht}}^{catab}\left(reht\right)\\ \;+k_{leak}\cdot \left(rcht-reht\right)-k_{rem}\cdot \left(reht\right)\\ \frac{d\left(rhia\right)}{dt}=V_{\text{rcht}}^{catab}\left(rcht\right)+V_{\text{reht}}^{catab}\left(reht\right)-k_{\text{rhia}}^{catab}\left(rhia\right)\\ \frac{d\left(rtryppool\right)}{dt}=\frac{400\cdot \left(rtryp\right)}{20+rtryp}-\left(0.6\right)\cdot \left(rtryppool\right)-k_{\text{rtryppool}}^{catab}\left(rtryppool\right) \end{array}$$

The DA terminal:

$$\begin{array}{l} \frac{d\left(dbh2\right)}{dt}=V_{\text{TH}}\left(dtyr,dbh4,dcda,deda\right)-V_{\text{DRR}}\left(dbh2,\text{NADPH},dbh4,\text{NADP}\right)\\ \frac{d\left(dbh4\right)}{dt}=V_{\text{DRR}}\left(dbh2,\text{NADPH},dbh4,\text{NADP}\right)-V_{\text{TH}}\left(dtyr,dbh4,dcda,deda\right)\\ \frac{d\left(dtyr\right)}{dt}=V_{\text{Lstyr}}\left(styr,styrp,sld\right)-V_{\text{TH}}\left(dtyr,dbh4,dcda,deda\right)\\ \;\;-k_{1}\cdot \left(dtyr\right)+k_{-1}\cdot \left(dtyrpool\right)-k_{dtyr}^{catab}\cdot \left(dtyr\right)\\ \frac{d\left(dld\right)}{dt}=V_{\text{Lld}}\left(sld,styr,stryp\right)+V_{\text{TH}}\left(dtyr,dbh4,dcda,deda\right)-V_{\text{dAADC}}\left(dld\right)\\ \frac{d\left(dcda\right)}{dt}=V_{\text{dAADC}}\left(dld\right)-V_{\text{dMAT}}\left(dcda,dvda\right)+V_{\text{DAT}}\left(deda\right)-k_{dcda}^{catab}\cdot \left(dcda\right)\\ \frac{d\left(dvda\right)}{dt}=V_{\text{MAT}}\left(dcda,dvda\right)-dfire\left(t\right)\cdot \left(dvda\right)\\ \frac{d\left(deda\right)}{dt}=dfire\left(t\right)\cdot \left(dvda\right)+\left(RFIRE\left(rinput\left(t\right),reht\right)\right)\cdot \left(5vda\right)-V_{\text{DAT}}\left(deda\right)\\ \;\;-V_{\text{deda}}^{catab}\left(deda\right)-k_{rem}\cdot \left(deda\right)\\ \frac{d\left(dhva\right)}{dt}=k_{dcda}^{catab}\cdot \left(dcda\right)+V_{\text{deda}}^{catab}\left(deda\right)-k_{dhva}^{catab}\cdot dhva\\ \frac{d\left(dtyrpool\right)}{dt}=k_{1}\cdot \left(dtyr\right)-k_{-1}\cdot \left(dtyrpool\right)-k_{dtyrpool}^{catab}\cdot \left(dtyrpool\right) \end{array}$$

Other:

$$\begin{array}{l} \frac{d\left(sld\right)}{dt}=lddose\left(t\right)-0.8\cdot \left(sld\right)\\ \frac{d\left(styr\right)}{dt}=0\\ \frac{d\left(stryp\right)}{dt}=0\\ \frac{d\left(5ld\right)}{dt}=V_{\text{Lsld}}\left(sld,styr,stryp\right)-V_{\text{AADC}5\text{ld}}\left(5ld,5htp\right)\\ \frac{d\left(5cda\right)}{dt}=V_{\text{AADC}5\text{ld}}\left(5ld,5htp\right)-V_{\text{MAT}5\text{cda}}\left(5cda,cht,5vda\right)-k_{5\text{cda}}^{catab}\cdot \left(5cda\right)\\ \frac{d\left(5vda\right)}{dt}=V_{\text{MAT}5\text{cda}}\left(5cda,cht,5vda\right)-\left(RFIRE\left(rinput\left(t\right),reht\right)\right)\cdot \left(5vda\right)\\ \frac{d\left(rld\right)}{dt}=V_{\text{Lsld}}\left(sld,styr,stryp\right)-V_{\text{AADCrld}}\left(rld,rhtp\right)\\ \frac{d\left(rcda\right)}{dt}=V_{\text{AADCrld}}\left(rld,rhtp\right)-V_{\text{MATrcda}}\left(rcda,rcht,rvda\right)-k_{\text{rcda}}^{catab}\cdot \left(rcda\right)\\ \frac{d\left(rvda\right)}{dt}=V_{\text{MATrcda}}\left(rcda,rcht,rvda\right)-\left(RFIRE\left(rinput\left(t\right),reht\right)\right)\cdot \left(rvda\right) \end{array}$$

### Discussion of key modeling issues

In the differential equations, some terms are given explicitly, but most velocities of enzymes or transporters are indicated by a function name starting with *V* followed (in parentheses) by the variables on which the velocity depends. The full model used in this paper combines our previous models of a dopamine terminal (Best et al., 2009) and a serotonin terminal (Best et al., 2010) with a new model for the serotonin cell body in the raphe nucleus, as well as new variables and differential equations for LD and DA in the 5HT cell body and terminal. In the absence of an LD dose, the steady state values for the variables in the DA and 5HT terminals are almost identical to the steady state values in Best et al. (2009, 2010). Except as indicated below, the functional forms of the velocities and the parameters in the terminals are exactly the same as in Best et al. (2009, 2010), and we refer the reader to those papers where detailed justifications are given.

Some reactions are given by the standard Michaelis–Menten formulas

$$\begin{array}{rc} V & =\frac{V_{max}\left[S\right]}{K_{m}+\left[S\right]},\;\;V=\frac{V_{max}\left[S_{1}\right]\left[S_{2}\right]}{\left(K_{S_{1}}+\left[S_{1}\right]\right)\left(K_{S_{2}}+\left[S_{2}\right]\right)}\\ V & =\frac{V_{max}^{f}\left[S_{1}\right]\left[S_{2}\right]}{\left(K_{S_{1}}+\left[S_{1}\right]\right)\left(K_{S_{2}}+\left[S_{2}\right]\right)}-\frac{V_{max}^{b}\left[P_{1}\right]\left[P_{2}\right]}{\left(K_{P_{1}}+\left[P_{1}\right]\right)\left(K_{P_{2}}+\left[P_{2}\right]\right)} \end{array}$$

for unidirectional, one substrate, unidirectional, two substrates, and bidirectional, two substrates, two products, reactions, respectively. Unless there are special issues, they are not discussed, but parameter values are given in Tables A2– A5, which contain all the parameters for the full model.

#### Competition for enzymes and transporters

One new element in the full model is the competition between DA, LD, and 5HT for the L-transporter, AADC, and VMAT, so we begin with a detailed discussion about how the competition is modeled. Consider the situation where an enzyme (or transporter) is in the presence of distinct substrates *S*_1_, *S*_2_,…, *S_N_*, which it turns into different products, *P*_1_, *P*_2_,…, *P_N_*. The enzyme has a different $K_{m}^{\left(i\right)}$ for each substrate and may have different dissociation constants, $K_{d}^{\left(i\right)}$ We’ll start with the case *N* = 2; the generalization will be obvious.

$$\begin{array}{r} S_{1}+E\overset{k_{1}}{\underset{k_{-1}}{⇆}}\;S_{1}\cdot E\overset{k_{d}^{\left(1\right)}}{\underset{}{\to }}\;P_{1}+E\\ S_{2}+E\overset{k_{2}}{\underset{k_{-2}}{⇆}}\;S_{2}\cdot E\overset{k_{d}^{\left(2\right)}}{\underset{}{\to }}\;P_{2}+E \end{array}$$

We make the equilibrium assumption, so:

$$\begin{array}{r} {\text{k}}_{1}\left[{\text{S}}_{1}\right]\left[\text{E}\right]={\text{k}}_{-1}\left[{\text{S}}_{1}\cdot \text{E}\right]\;and\;{\text{k}}_{2}\left[{\text{S}}_{2}\right]\left[\text{E}\right]={\text{k}}_{-2}\left[{\text{S}}_{2}\cdot \text{E}\right]. \end{array}$$

Define *E_tot_* = [*E*] + [*S*_1_·*E*] + [*S*_2_·*E*] Then,

$$\begin{array}{l} V_{1}=\frac{dP_{1}}{dt}=k_{d}^{\left(1\right)}\left[S_{1}\cdot E\right]=\frac{k_{4}E_{tot}\left(\frac{k_{1}}{k_{-1}}\right)\left[S_{1}\right]\left[E\right]}{E_{tot}}\\ =\frac{k_{d}^{\left(1\right)}E_{tot}\left(\frac{k_{1}}{k_{-1}}\right)\left[S_{1}\right]\left[E\right]}{\left[E\right]+\left[S_{1}\cdot E\right]+\left[S_{2}\cdot E\right]}\\ =\frac{k_{d}^{\left(1\right)}E_{tot}\left(\frac{k_{1}}{k_{-1}}\right)\left[S_{1}\right]\left[E\right]}{\left[E\right]+\left(\frac{k_{1}}{k_{-1}}\right)\left[S_{1}\right]\left[E\right]+\left(\frac{k_{2}}{k_{-2}}\right)\left[S_{2}\right]\left[E\right]}\\ =\frac{k_{d}^{\left(1\right)}E_{tot}\left[S_{1}\right]}{\left(\frac{k_{-1}}{k_{1}}\right)+[S_{1}][E]+\left(\frac{k_{-1}}{k_{1}}\right)\left(\frac{k_{2}}{k_{-2}}\right)[S_{2}]}\\ =\frac{k_{d}^{\left(1\right)}E_{tot}[S_{1}]}{K_{m}^{\left(1\right)}\left(1+\frac{\left[s_{2}\right]}{k_{m}^{\left(2\right)}}\right)+S_{1}}, \end{array}$$

where we define $K_{m}^{\left(i\right)}=\left(k_{-i}∕k_{i}\right)$. Thus the general formula is:

(A1) $$V_{i}=\frac{k_{d}^{\left(1\right)}E_{tot}[S_{i}]}{K_{m}^{\left(i\right)}\left(1+\sum_{j\neq i}\frac{\left[S_{j}\right]}{K_{m}^{\left(j\right)}}\right)+[S_{i}]},$$

which is just the formula in Smith’s article in Kilberg and Haussinger (1992). Note that the effect of the competition is to raise the effective *K_m_* of each amino acid.

When an enzyme has different substrates with different velocities, we indicate which velocity by including the substrate name after the name of the enzyme or transporter. Thus, *V*_Lstyr_, *V*_Lstryp_, and *V*_Lsld_, indicate the velocities at which *styr*, *stryp*, and *sld* are transported into the brain by the L-transporter. Each of these velocities depends on all three variables as shown in formula (1). We note that the BBB is actually quite complex, consisting of two layers of cells, and that once substrates have passed they are in the extracellular fluid and will compete again for transport into cells. We simplify this complex process by having a single competitive transport process from the serum directly into brain cells.

During an LD dose, 5*ld* and 5*ht* compete for AADC in the 5HT terminal, and these velocities are indicated by *V*_AADC5htp_, *V*_AADC5ld_. In the raphe cell body the velocities are indicated by *V*_AADCrhtp_, *V*_AADCrld_. And, in the 5HT terminal and raphe cell body, DA and 5HT compete for the vesicular monoamine transporter, MAT, and the nomenclature is analogous.

#### The model for the dorsal raphe cell body and the 5HT1a autoreceptor

The second new feature of the full model is a new model for the cell body of the 5HT neuron in the dorsal raphe nucleus. Most of the model biochemistry in the cell body is identical to the model biochemistry in the terminal in Best et al. (2010). The exceptions are the competition discussed above, changed parameters for MAT (discussed below), and the 5HT1a autoreceptors of the cell body. These 5HT neurons have the unusual property that when the cell fires an action potential, 5HT is not only released at the synapse but also is released from varicosities and the cell body (Adell et al., 2002). In addition, simple diffusion of 5HT from the cytosol to the extracellular space accounts for approximately 30% of the released 5HT at the cell body (Adell et al., 2002). Therefore, we include a simple linear diffusion term in the differential equations for *rcht* and *reht* with rate constant *k_leak_* chosen so that the leakage release is 30% of total release at the cell body when the neuron is firing tonically at 1 Hz.

Extracellular 5HT (reht) at the cell body binds to 5HT1a auroreceptors. As *reht* goes up, the synthesis rate, *V*_TPH_ declines. We use the same functional form for this inhibition as in Best et al. (2010) for the way that the 5HT1B autoreceptors affect synthesis in the terminal. As *reht* goes up the 5HT1a autoreceptors cause the firing rate to decline. We model this with the formula:

$$\text{RFIRE}\left(reht\right)=1.5\;-\;\frac{{\left(reht\right)}^{2}}{\left({\left(.001\right)}^{2}+{\left(reht\right)}^{2}\right)},$$

so that when *reht* equals 1 nanomolar (a typical concentration) the firing rate is 1 Hz and when *reht* goes up firing can become as slow as 0.5 Hz. We note that the 5HT cell body affects the 5HT terminal through the term RFIRE.

The effect of *eht* in the striatum on synthesis and release in the 5HT terminal is modeled as in Best et al. (2010). The effect of *eda* in the striatum on DA synthesis via the DA autoreceptors is modeled as in Best et al. (2009).

#### Serum concentrations of tyr, tryp, and LD

For the full model we take the serum concentrations to be *styr* = 63 μM and *stryp* = 81 μM as given in Kilberg and Haussinger (1992). We adjusted the *V_max_* values so that the fluxes into the brain (in the absence of an LD dose) are *V_Ltyr_* = 248 μM/h and *V_Ltryp_* = 151 μM/h as found in Kilberg and Haussinger (1992). The LD dose in the serum is modeled as indicated in Figure 1. The half-life of the dose is approximately 90 min as is found experimentally (Khor and Hsu, 2007), and we choose the size of the dose so the serum concentration of LD is comparable to the serum concentrations of *tyr* and *tryp* consistent with the clinical and experimental literature (Morris et al., 1976; Rinne and Molsa, 1979; Hutton et al., 1988; Meuller et al., 2005; Khor and Hsu, 2007). We note that the actual LD dose in the serum depends on variable transport from the gut and the presence or absence of an AADC inhibitor, as well the size of the dose itself. Not surprisingly, the size of changes seen in the model during an LD dose (for example, the drop of *eht* in the striatum) depends on the size of the dose.

#### The blood-brain barrier

We take the *K_m_* of the L-transporter for *tyr* to be 64 μM and the *K_m_* for *tryp* to be 15 μM as reported in Kilberg and Haussinger (1992). There are a wide range of values, 10–102 μM for the *K_m_* for transport of LD into different types of cells (Sampaio-Maia et al., 2001; Hu and Li, 2011). We chose a value, 32 μM, in the lower part of this range. The formulas for *V*_Lstyr_, *V*_Lstryp_, and *V*_Lsld_ are given by formula (1) with the two unnamed substrates being the competitors.

#### AADC

For aromatic amino acid decarboxylase, we take the *K_m_* = 160 M for 5*htp* (Sumi et al., 1990) and the *K_m_* = 130 for *ld* (Siaterli et al., 2003). These are the same values used in Best et al. (2009, 2010). Thus, using formula (1), the velocity by which *cda* is made from 5*ld* in the 5HT terminal is

$$V_{5\text{dAADC}}\left(5ld,5htp\right)=\frac{V_{max}^{\left(1\right)}\left(5ld\right)}{\left(130\right)(1+\frac{5htp}{160})+\left(5ld\right)},$$

and the velocity by which *cht* is made from 5*htp* in the 5HT terminal is

$$V_{5\text{AADC}}\left(5htp,5ld\right)=\frac{V_{max}^{\left(2\right)}\left(5htp\right)}{\left(160\right)(1+\frac{5ld}{130})+\left(5htp\right)}.$$

#### MAT

For the monoamine transporter, we take the *K_m_* = 0.2 μM for 5*ht* (Slotkin et al., 1978; Rau et al., 2006) and the *K_m_* = 3 μM for *cda* (Sherman and Henry, 1983; Near, 1986; Volz et al., 2006). These are the same values used in Best et al. (2009, 2010). Thus, thenet velocity at which *cda* is packaged into vesicles in the 5HT terminal is

$$V_{\text{MAT}5\text{cda}}\left(5cda,5ht\right)=\frac{V_{max}^{\left(1\right)}\left(cda\right)}{\left(3\right)(1+\frac{\left(5ht\right)}{3})+\left(5cda\right)}\;-\;k_{out}\left(5vda\right),$$

and the net velocity at which 5*ht* is packaged into vesicles in the 5HT terminal is

$$V_{\text{MATcht}}\left(5ht,5cda\right)=\frac{V_{max}\left(5ht\right)}{\left(0.2\right)(1+\frac{\left(5cda\right)}{.2})+\left(5ht\right)}\;-\;k_{out}\left(5vht\right).$$

The terms −*k_out_*(5*vda*) and −*k_out_*(5*vda*) represent backleak out of the vesicles as described in Floor et al. (1995) and Wallace (2007) and here is the only substantial difference between the parameters in the full model for this paper and the parameters for the two terminal models, Best et al. (2009, 2010). The net flux into the vesicular compartment and the release into the extracellular space determine the concentration (of 5HT or DA) in the cytosolic and vesicular compartments. Since we had a good idea about those concentrations, we knew well what the net flux ought to be. However, we had no way a gaging what the in and out fluxes should be. When we started experimenting with the full model in this paper, the LD dose stimulated instantaneous leakage fluxes that were unphysiological. We therefore lowered both the *V_max_* in the forward direction and the *k_out_* in the backward direction substantially. All parameters for the full model can be found in Tables A2– A5.

## References

[B86] Armarego W. Ohnishi A. Taguchi H. (1986). New pteridine substrates for dihydropteridine reductase and horseradish peroxidase. Biochem. J. 234, 335–342 3718470 10.1042/bj2340335 PMC1146570

[B87] Bailey S. Ayling J. (1983). 6,6-Dimethylpterins: stable quinoid dihydropterin substrate for dihydropteridine reductase and tetrahydropterin cofactor for phenylalanine hydroxylase. Biochemistry 22, 1790–1798 10.1021/bi00277a008 6849887

[B88] Best J. A. Nijhout H. F. Reed M. C. (2010). Serotonin synthesis, release and reuptake in terminals: a mathematical model. Theor. Biol. Med. Model. 7, 34 10.1186/1742-4682-7-34 20723248 PMC2942809

[B89] Bunin M. Prioleau C. Mailman R. Wightman R. (1998). Release and uptake rates of 5-hydroxytryptamine in the dorsal raphe and substantia nigra of the rat brain. J. Neurochem. 70, 1077–1087 10.1046/j.1471-4159.1998.70031077.x 9489728

[B90] Craine J. Hall E. Kaufman S. (1972). The isolation and characterization of dihydropteridine reductase from sheep liver. J. Biol. Chem. 247, 6082–6091 4405600

[B91] Daws L. Montenez S. Owens W. Gould G. Frazer A. Toney G. Gerhardt G. (2005). Transport mechanisms governing serotonin clearance in vivo revealed by high speed chronoamperometry. J. Neurosci. Methods 143, 49–62 10.1016/j.jneumeth.2004.09.011 15763136

[B92] Dedek J. Baumes R. Tien-Duc N. Gomeni R. Korf J. (1979). Turnover of free and conjugated (sulphonyloxy) dihydrophenylacetic acid and homovanillic acid in rat striatum. J. Neurochem. 33, 687–695 10.1111/j.1471-4159.1979.tb05213.x 479883

[B93] Echizen H. Freed C. R. (1984). Measurement of serotonin turnover rate in rat dorsal raphe nucleus by in vivo electrochemistry. J. Neurochem. 42, 1483–1486 10.1111/j.1471-4159.1984.tb02815.x 6200572

[B94] Firgaira F. Cotton R. Danks D. (1981). Isolation and characterization of dihydropteridine reductase from human liver. Biochem. J. 97, 31–43 7317032 10.1042/bj1970031 PMC1163052

[B95] Firgaira F. Cotton R. Jennings I. Danks D. (1987). Use of naphthoquinone adsorbant for the isolation of human dihydropteridine reductase. Meth. Enzymol. 142, 116–126 10.1016/S0076-6879(87)42018-1 3600366

[B96] Fowler C. J. Ross S. B. (1984). Selective inhibitors of monoamine oxidase a and b: biochemical, pharmacological, and clinical properties. Med. Res. Rev. 4, 323–358 10.1002/med.2610040303 6379342

[B97] Gottowik J. Cesura A. M. Malherbe P. Lang G. Prada M. D. (1993). Characterisation of wild-type and mutant forms of human monoamine oxidase a and b expressed in a mammalian cell line. FEBS Lett. 317, 152–156 10.1016/0014-5793(93)81512-X 8428624

[B98] Hu M. Li X. (2011). Oral Bioavailability: Basic Principles, Advanced Concepts, and Applications. New York: Wiley

[B99] Hutton J. T. Albrecht J. W. Roman G. C. Kopetzky M. T. (1988). Prolonged serum levodopa levels with controlled-release carbidopa-levodopa in the treatment of Parkinson’s disease. Arch. Neurol. 45, 55–57 10.1001/archneur.1988.00520320047014 3276299

[B100] Jones S. Garris P. Kilts C. Wightman R. (1995). Comparison of dopamine uptake in the basolateral amygdaloid nucleaus, caudate-putamen, and nucleus accumbems of the rat. J. Neurochem. 64, 2581–2589 10.1046/j.1471-4159.1995.64062581.x 7760038

[B101] Karoum F. Norton H. Wyatt R. (1977). The dynamics of dopamine metabolism in various regions of rat brain. Eur. J. Pharmacol. 44, 311–318 10.1016/0014-2999(77)90304-1 891608

[B102] McKinney J. Knappskog P. M. Haavik J. (2005). Different properties of the central and peripheral forms of human tryptophan hydroxylase. J. Neurochem. 92, 311–320 10.1111/j.1471-4159.2004.02850.x 15663479

[B103] Meuller T. Fowler B. Kuhn W. (2005). Levodopa intake increases plasma levels of s-adenosylmethionine in treated patients with Parkinson disease. Clin. Neuropharmacol. 28, 274–276 10.1097/01.wnf.0000190800.87380.c7 16340382

[B104] Morgenroth V. Walters J. Roth R. (1976). Dopaminergic neurons – alteration in the kinetic properties of tyrosine hydroxylase after cessation of impulse flow. Biochem. Pharmacol. 25, 655–661 10.1016/0006-2952(76)90240-9 6035

[B105] Morris J. Parsons R. L. Trounce J. R. Groves M. J. (1976). Plasma dopa concentrations after different preparations of levodopa in normal subjects. Br. J. Clin. Pharmacol. 3, 985–990 10.1111/j.1365-2125.1976.tb00347.x PMC1428975 22216519

[B106] Nakashima A. Mori K. Suzuki T. Kurita H. Otani M. Nagatsu T. Ota A. (1999). Dopamine inhibition of human tyrosine hydroxylase type i is controlled by the specific portion of the n-terminus of the enzyme. J. Neurochem. 72, 2145–2153 10.1046/j.1471-4159.1999.0722145.x 10217296

[B107] Near J. (1986). [3h]Dihydrotetra-benazine binding to bovine striatal synaptic vesicles. Mol. Pharmacol. 30, 252–257 3748008

[B108] Rau K. S. Birdsall E. Volz T. J. Riordan J. A. Baucum A. J. Adair B. P. Bitter R. Gibb J. W. Hanson G. R. Fleckenstein A. E. (2006). Methamphetamine administration reduces hippocampal vesicular monoamine transporter-2 uptake. J. Pharmacol. Exp. Ther. 318, 676–682 10.1124/jpet.105.099200 16687477

[B109] Rinne U. K. Molsa P. (1979). Levodopa with benserazide or carbidopa in Parkinson’s disease. Neurology 29, 1584–1589 10.1212/WNL.29.12.1584 574221

[B110] Rivett A. Roth J. (1982). Kinetic studies on the o-methylation of dopamine by human brain membrane-bound catechol o-methyltransferase. Biochemistry 21, 1740–1742 10.1021/bi00537a006 7082642

[B111] Royo M. Daubner S. Fitzpatrick P. (2004). Effects of mutations in tyrosine hydroxylase associated with progressive dystonia on the activity and stability of the protein. Proteins 58, 14–21 10.1002/prot.20293 15468323 PMC1945158

[B112] Sampaio-Maia B. Serrao M. P. da Silva P. S. (2001). Regulatory pathways and uptake of l-dopa by capillary cerebral endothelial cells, astrocytes, and neuronal cells. Am. J. Physiol. Cell Physiol. 280, C333–C342 11208529 10.1152/ajpcell.2001.280.2.C333

[B113] Schmitz Y. Benoit-Marand M. Gonon F. Sulzer D. (2003). Presynaptic regulation of dopaminergic neurotransmission. J. Neurochem. 87, 273–289 10.1046/j.1471-4159.2003.02050.x 14511105

[B114] Schomburg D. Schomburg I. (2005). “6,7-Dihydropteridine reductase,” in Springer Handbook of Enzyme, Vol. 23, (Berlin: Springer) 248–272

[B115] Sherman D. Henry J. (1983). The catecholamine carrier of bovine Chromaffin granules. Mol. Pharmacol. 23, 431–436 6835201

[B116] Siaterli M. Vassilacopoulou D. Fragoulis E. (2003). Cloning and expression of human placental l-dopa decarboxylase. Neurochem. Res. 28, 797–803 10.1023/A:1023246620276 12718431

[B117] Slotkin T. A. Seidler F. J. Whitmore W. L. Lau C. Salvaggio M. Kirksey D. K. (1978). Rat brain synaptic vesicles: uptake specificities of [3h]norepinephrine and [3h]serotonin in preparations from whole brain and brain regions. J. Neurochem. 31, 961–968 10.1111/j.1471-4159.1978.tb00134.x 702157

[B118] Sumi C. Ichinose H. Nagatsu T. (1990). Characterization of recombinant human aromatic l-amino acid decarboxylase expressed in cos cells. J. Neurochem. 55, 1075–1078 10.1111/j.1471-4159.1990.tb04601.x 2117047

[B119] Volz T. Hanson G. Fleckenstein A. (2006). Kinetic analysis of developmental changes in vesicular monoamine transporter-2 function. Synapse 60, 474–477 10.1002/syn.20321 16897727

